# PLGA-Based Strategies for Intranasal and Pulmonary Applications

**DOI:** 10.3390/pharmaceutics17020207

**Published:** 2025-02-06

**Authors:** Hossein Omidian, Renae L. Wilson

**Affiliations:** Barry and Judy Silverman College of Pharmacy, Nova Southeastern University, Fort Lauderdale, FL 33328, USA

**Keywords:** PLGA drug delivery, intranasal applications, pulmonary drug delivery, sustained release systems, biocompatible nanoparticles

## Abstract

Poly(D,L-lactide-co-glycolide) (PLGA) has emerged as a cornerstone in the development of advanced drug delivery systems, particularly for intranasal and pulmonary routes. Its biodegradability, biocompatibility, and adaptability make it an ideal platform for addressing challenges associated with conventional therapies. By enabling sustained and controlled drug release, PLGA formulations reduce dosing frequency, improve patient compliance, and enhance therapeutic efficacy. These systems demonstrate versatility, accommodating hydrophilic and hydrophobic drugs, biological molecules, and co-delivery of synergistic agents. Moreover, surface modifications and advanced preparation techniques enhance targeting, bioavailability, and stability, expanding PLGA’s applications to treat complex diseases such as tuberculosis, cancer, pulmonary fibrosis, and CNS disorders. This manuscript provides an in-depth review of PLGA’s materials, properties, preparation methods, and therapeutic applications, alongside a critical evaluation of challenges and future opportunities in this field.

## 1. The Role of PLGA in Enhancing Nasal and Pulmonary Drug Delivery Systems

The development of effective drug delivery systems remains a cornerstone of modern pharmaceutical science, particularly for challenging administration routes like nasal and pulmonary pathways. These routes offer significant therapeutic potential, providing direct access to systemic circulation or target sites such as the central nervous system (CNS) and respiratory tract while bypassing barriers like first-pass metabolism or the blood–brain barrier. The application of PLGA, a biodegradable and biocompatible polymer, has transformed intranasal and pulmonary drug delivery by enabling sustained release, enhanced targeting, and reduced systemic toxicity [1,2,3,4].

PLGA’s unique properties, including its ability to degrade into non-toxic byproducts such as lactic and glycolic acids, make it a safe and versatile material for long-term applications in sensitive tissues like the nasal mucosa and lungs [1,5,6,7]. Moreover, its adaptability to a wide range of preparation techniques, such as emulsification, Flow Focusing^®^ technology, spray drying, and supercritical fluid processing, allows for the tailored design of drug delivery systems to meet specific therapeutic requirements [2,5,8,9,10].

Intranasal delivery using PLGA offers a non-invasive approach to accessing the CNS, leveraging the olfactory and trigeminal pathways for effective treatment of neurodegenerative disorders and brain cancers [11,12,13]. Similarly, pulmonary delivery systems using PLGA enable deep lung deposition and retention, which are crucial for treating diseases such as tuberculosis, asthma, and pulmonary hypertension [5,14,15,16]. The ability of PLGA to encapsulate a wide range of drugs, including hydrophilic, hydrophobic, and biological molecules, further underscores its versatility and relevance in modern drug delivery [10,17,18,19].

Additionally, PLGA-based systems have demonstrated compatibility with advanced surface modifications, such as ligand conjugation and chitosan coating, to enhance targeting, mucoadhesion, and bioavailability for both systemic and localized therapies [3,12,20,21,22]. The incorporation of cryoprotectants and stabilizers ensures the long-term stability of these formulations, enabling practical storage and transportation for commercial use [23,24,25]. Furthermore, the ability to co-deliver multiple therapeutic agents expands the potential of PLGA in treating complex diseases like cancer and cystic fibrosis through synergistic effects [15,26,27].

The application of PLGA in intranasal and pulmonary drug delivery presents a promising strategy to overcome the limitations of conventional therapies by enabling targeted, sustained, and innovative solutions for localized, systemic, and CNS-directed treatments. Its biocompatibility, adaptability across diverse drug formulations, and potential to address challenges such as poor bioavailability and rapid clearance have made PLGA an essential platform for treating CNS disorders, respiratory diseases, and other complex conditions. However, issues such as inconsistent drug release, physiological variability, and safety concerns related to functionalized particles underscore the need for further innovation in polymer modification, surface engineering, and production standardization. Refining formulation stability and ensuring long-term safety are critical to achieving successful clinical translation.

## 2. Therapeutic Applications of PLGA in Nasal and Pulmonary Route

### 2.1. Enhancing Antibiotic Therapies for Respiratory Infections

PLGA-based formulations are highly effective in enhancing antibiotic therapies for respiratory infections, providing prolonged release and reducing dosing frequency. Levofloxacin and tobramycin are examples of antibiotics designed for sustained pulmonary release, extending therapeutic action and providing ongoing protection against bacterial pathogens in chronic respiratory conditions [28,29]. Importantly, safety studies have shown that celecoxib-loaded PLGA microparticles caused no pulmonary toxicity during extended evaluations, as confirmed by histological and biochemical analyses over 21 days [8]. Similarly, testing on lung cell lines exposed to PLGA at concentrations as high as 10 mg/mL demonstrated no cytotoxicity, further validating its suitability for antibiotic delivery in respiratory therapies [4].

Similarly, ethionamide and ciprofloxacin, encapsulated in PLGA for inhalation, deliver extended antimicrobial effects, which is particularly valuable in managing persistent and severe respiratory infections [30,31]. Cryoprotectants like sucrose are often employed during the freeze-drying of PLGA nanoparticles, reducing aggregation and maintaining the stability of these antibiotic formulations, thus preserving their physicochemical properties [32]. This sustained-release approach not only maintains consistent therapeutic levels but also improves patient adherence, potentially reducing the risk of antibiotic resistance.

### 2.2. Anti-Inflammatory and Anti-Fibrotic Applications

PLGA-based systems are also used for delivering anti-inflammatory and anti-fibrotic drugs, particularly in treating chronic lung diseases. Encapsulation of alpha-mangostin, an anti-inflammatory compound, in PLGA nanoparticles ensures prolonged pulmonary delivery, making it suitable for managing inflammation in chronic respiratory conditions [17]. Drugs like celecoxib, nintedanib, and pirfenidone, often formulated in PLGA nanoparticles or combined with liposomes, provide targeted therapy for pulmonary fibrosis, where fibrotic lung tissue necessitates extended and controlled drug release [8,33]. Importantly, advanced formulations such as porous PLGA microparticles have been engineered to evade macrophage uptake, minimizing off-target effects and increasing local lung retention [34]. These biocompatibility improvements ensure that PLGA-based systems deliver anti-inflammatory and anti-fibrotic drugs effectively without triggering significant adverse effects.

### 2.3. Combination Therapies for Complex Respiratory Conditions

**Figure 1:**
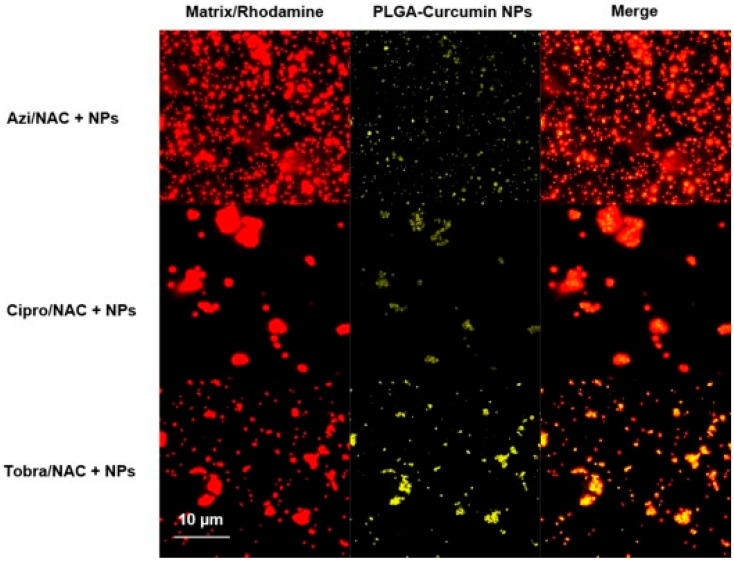
Shows the morphology of multidrug inhalable microparticles using confocal laser scanning microscopy (CLSM): matrix/rhodamine B fluorescence (**left row**), the fluorescence of the curcumin nanoparticles (**second row**) and both images merged (**right row**). The yellow indicates the superposition of red and green color in the respective areas. Adopted with permission [15].

In complex respiratory conditions, combination therapies using PLGA are particularly advantageous. For example, a formulation combining tobramycin, ciprofloxacin, azithromycin, N-acetylcysteine (NAC), and curcumin addresses infections, mucus buildup, and inflammation simultaneously, highlighting PLGA’s versatility in multimodal respiratory treatment (Figure 1) [15]. In addition to their therapeutic benefits, PLGA nanoparticles demonstrate excellent safety in combination therapy contexts, with minimal oxidative stress or hemolysis observed in preclinical studies [13].

### 2.4. PLGA-Based Pulmonary Delivery of Peptides and Proteins

**Figure 2:**
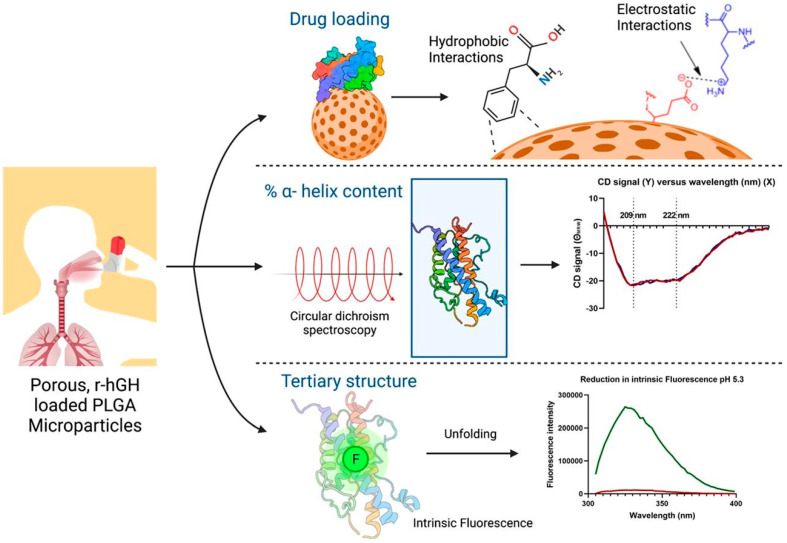
Schematic development of an r-hGH-loaded PLGA and PEG-based porous microparticles as vehicles for pulmonary somatropin delivery over a period of seven days. Adopted with permission [6].

Peptide and protein drugs, traditionally challenging to administer, also benefit from PLGA-based pulmonary and systemic delivery systems. Insulin, developed for pulmonary delivery using PLGA, presents a promising non-invasive option for diabetes management with extended-release profiles [35,36]. Similarly, respiratory therapies leverage PLGA for delivering therapeutic proteins like Deoxyribonuclease I (DNaseI), which breaks down extracellular DNA in lung secretions to improve respiratory function in cystic fibrosis by enhancing mucus clearance [37]. Proteins such as alpha-1 antitrypsin and somatropin (r-hGH) are also formulated with PLGA for targeting inflammatory and degenerative lung conditions, providing protein stability and controlled release for chronic treatments requiring long-term efficacy [38]. Figure 2 shows a schematic preparation of large, porous, biodegradable, aerodynamically light microparticles as tunable carriers for pulmonary r-hGH delivery [6]. These formulations have been tested for biocompatibility, demonstrating no significant cytotoxicity or inflammatory reactions, which is crucial for maintaining the safety of prolonged protein therapies.

### 2.5. Innovations in Nanoparticle Design for Biologics

The FDA’s regulatory approval of PLGA has also enabled innovation in nanoparticle design for biologics. For example, recombinant human interleukin-2 (rhIL-2)-loaded PLGA microparticles for pulmonary administration demonstrated high encapsulation efficiency and retained biological activity post-encapsulation [39]. These findings further underscore PLGA’s potential as an effective carrier for biologics, expanding its therapeutic applications while leveraging its established safety profile.

### 2.6. Intranasal PLGA-Based Formulations for CNS Drug Delivery

Intranasal PLGA-based formulations have shown significant potential in delivering neurological drugs directly to the brain, bypassing the blood–brain barrier and enabling rapid therapeutic action. Chlorpromazine hydrochloride, for instance, is formulated for CNS applications via nasal administration, with PLGA supporting controlled drug delivery and efficient brain targeting [20]. Dopamine agonists such as rotigotine and ropinirole hydrochloride, used in Parkinson’s disease treatment, are encapsulated in PLGA for intranasal delivery, enabling precise brain targeting and controlled drug release [12,13]. These formulations benefit from PLGA’s proven safety profile in the CNS context, as evidenced by studies where lactoferrin-modified PLGA nanoparticles showed enhanced brain targeting without inducing toxicity or systemic side effects [40]. Similarly, antiepileptic drugs like oxcarbazepine and thyrotropin-releasing hormone analogs (NP-355, NP-647) are effectively delivered through the nose-to-brain route, leveraging PLGA’s capabilities for localized delivery, minimal systemic exposure, and rapid onset of therapeutic effects [41,42].

### 2.7. Oncology Applications of PLGA in Lung and Brain Cancers

The role of PLGA in oncology highlights its effectiveness in achieving targeted delivery and reducing adverse systemic effects, particularly for lung and brain cancers. For lung cancer, anticancer agents like doxorubicin (DOX), cisplatin, and docetaxel are incorporated into PLGA formulations for controlled pulmonary delivery, enhancing drug targeting to tumor sites and reducing systemic toxicity [26,43]. Artesunate-loaded PLGA microparticles, for example, demonstrated excellent safety profiles while inducing apoptosis and inhibiting cancer cell migration and invasion [43]. In CNS applications, intranasal PLGA-based formulations for drugs like docetaxel and curcumin target brain tumors such as glioblastoma, achieving localized therapeutic action, reducing side effects, and potentially increasing efficacy in aggressive brain cancers [32,44].

PLGA-based formulations have demonstrated remarkable versatility in advancing therapeutic applications through nasal and pulmonary routes, addressing critical challenges such as sustained drug release, patient adherence, and targeted delivery. However, limitations including burst-release effects, protein instability, macrophage clearance, and challenges in multi-drug encapsulation must be systematically addressed to fully exploit the potential of PLGA systems. Regulatory approval of PLGA provides a solid foundation for clinical translation, but precise optimization of manufacturing processes, drug loading, and stability remains pivotal. With advancements in surface functionalization, cryoprotectant utilization, and scalable techniques, PLGA formulations are poised to transform the management of complex diseases such as chronic respiratory infections, CNS disorders, and cancers.

Table 1 explores the therapeutic applications of various drugs encapsulated in PLGA formulations, focusing on pulmonary, CNS, and systemic delivery. It highlights key drug groups and their roles in treating infections, cancer, fibrosis, and neurodegenerative diseases, showcasing the versatility of PLGA-based systems. PLGA formulations enable a broad spectrum of therapeutic uses, including sustained drug delivery for chronic infections, tumor targeting for lung and CNS cancers, and enhanced brain targeting for neurodegenerative diseases. The versatility of PLGA systems supports tailored treatments for diseases like pulmonary fibrosis, tuberculosis, and hypertension. Synergistic drug combinations (e.g., docetaxel and celecoxib) improve efficacy and reduce side effects. Advanced delivery mechanisms for hormonal, protein, and gene therapies indicate the potential of PLGA for precision medicine.

## 3. Key Materials Supporting PLGA Formulations in Intranasal and Pulmonary Delivery

PLGA polymers serve as the foundation for many intranasal and pulmonary drug delivery systems, widely valued for their biocompatibility and biodegradability. PLGA safely degrades into lactic and glycolic acids, which are naturally metabolized and eliminated through the Krebs cycle, ensuring minimal toxicity and patient safety across various therapeutic applications [1]. To enhance mucoadhesion and prolong retention at mucosal surfaces, chitosan and its derivatives, such as N-trimethyl chitosan (TMC), are often incorporated [76]. Chitosan’s adhesive properties improve retention at nasal sites, making it advantageous for formulations requiring sustained presence, such as nasal vaccines, by promoting prolonged antigen exposure [20,28,71].

### 3.1. Improving Stability and Circulation with PEG and PVA

Poly(ethylene glycol) (PEG) and PEG-PLGA copolymers play crucial roles in improving stability and reducing macrophage uptake, extending circulation time in the bloodstream. This extended half-life is particularly beneficial for formulations aimed at brain delivery, where prolonged systemic exposure aids in effective CNS penetration [12,80]. Polyvinyl alcohol (PVA) acts as a stabilizing agent essential for maintaining particle integrity, particularly in nanoparticle formulations. Its use enhances particle formation and encapsulation efficiency, leading to consistent drug release—a critical factor for both nasal and pulmonary applications [17,58].

### 3.2. Optimizing Aerosolization with Lactose, Sorbitol, and Surfactants

Lactose and sorbitol are commonly used as dispersing agents to improve aerosolization properties. Lactose, often employed in dry powder inhalers (DPIs), enhances powder flow and dispersion, while sorbitol supports the uniform dispersion of PLGA particles, optimizing pulmonary delivery efficiency [30,81]. Surfactants like Kolliphor^®^ P 188 help control particle size for consistent distribution in inhalable formulations, improving aerodynamic properties necessary for effective pulmonary delivery [7,82].

### 3.3. Specialized Ligands for CNS Targeting

Specialized ligands are used for formulations targeting the central nervous system (CNS), enhancing targeting and penetration of the blood–brain barrier (BBB). Lactoferrin facilitates receptor-mediated transport, optimizing delivery efficiency in neurodegenerative treatments, while lactoferrin-conjugated N-trimethyl chitosan (Lf-TMC) further enhances cellular uptake, advancing nasal-to-brain delivery for neurological conditions [12,40]. Other agents, such as odorranalectin (OL) and Rabies Virus Glycoprotein (RVG29), are effective in crossing the BBB via receptor-mediated uptake, improving CNS delivery in therapies for Alzheimer’s and epilepsy [83,84]. Polysorbate 80, a surfactant used to coat nanoparticles, increases brain uptake by enhancing membrane permeability, making it particularly useful in neurotherapies [64].

### 3.4. Porogens and Agents for Controlled Release and Cellular Uptake

Controlled release and enhanced cellular uptake in formulations often rely on porogens like ammonium bicarbonate (ABC) and sodium bicarbonate to create porous microparticles. The resulting porosity improves dispersion and allows for controlled drug release profiles, especially useful in pulmonary and intranasal applications [18,34]. Polyethyleneimine (PEI), a strong cationic agent, is frequently employed in DNA and siRNA delivery systems to facilitate cellular uptake and enhance transfection efficiency, a key attribute in gene therapy formulations [59,72]. Poloxamer 407 and HPMC K4M further enhance mucoadhesion, extending residence time within the nasal cavity to improve brain-targeting efficiency, which is particularly beneficial for treating neurological conditions [65].

### 3.5. Immune-Boosting Adjuvants for Vaccines

For vaccine applications, immune response is significantly boosted with specific adjuvants. Quillaja saponins (QS-21) and cross-linked dextran microspheres (CDM) are used in PLGA-based vaccine formulations to promote immune responses by enhancing antigen presentation. This increased antigenicity supports the development of effective nasal vaccines, which benefit from prolonged immune system interaction at the administration site [85]. Calcium phosphate is employed as a protein-stabilizing agent in self-healing PLGA microspheres, maintaining antigenicity and allowing for prolonged antigen release, which significantly enhances the efficacy of vaccine delivery systems [73].

### 3.6. Structural and Functional Enhancers in PLGA-Based Formulations

To improve structural stability and delivery efficiency, PLGA-based formulations incorporate various structural and functional enhancers. Poly(methyl vinyl ether-co-maleic anhydride) (PVM/MA), a mucoadhesive matrix, increases residence time in the lungs, providing prolonged therapeutic impact in pulmonary applications [86]. Hydroxypropyl-beta-cyclodextrin (HPbetaCD) enhances the solubility and bioavailability of encapsulated molecules, enabling sustained release in conditions such as pulmonary hypertension [60]. Additionally, bovine serum albumin serves a stabilizing role in formulations designed for pH-dependent release, creating core–shell structures that offer particle stability and support sensitive biomolecules [7].

The materials supporting PLGA formulations for intranasal and pulmonary drug delivery are critical to optimizing therapeutic efficacy, stability, and targeting. Enhancers such as chitosan derivatives, PEG copolymers, and surfactants improve mucoadhesion, circulation time, and aerodynamic properties, respectively, addressing key challenges in these delivery routes. However, issues such as variability in mucoadhesive strength, immunogenicity of PEG, and residual stabilizer content remain barriers to achieving consistent outcomes. Ligand-based targeting systems and porogen-induced porosity enhance precision and controlled release but face challenges related to receptor heterogeneity and potential cytotoxicity. Similarly, adjuvants like QS-21 and stabilizers such as calcium phosphate significantly improve vaccine efficacy but require careful balancing to minimize inflammatory risks. Future advancements should focus on developing hybrid systems and refining characterization methods to achieve reproducible and scalable formulations while minimizing adverse effects.

Table 2 highlights the roles of PLGA, along with other polymers and excipients, in optimizing drug delivery systems. It emphasizes their impact on bioavailability, mucoadhesion, stability, and targeted delivery for pulmonary, nasal, and CNS applications. PLGA is frequently combined with excipients like chitosan, PVA, and cyclodextrins to enhance mucoadhesion, stability, and bioavailability. Poloxamers and amphiphilic copolymers contribute to tunable aerodynamic and physical properties for pulmonary applications. PEI and other modifiers improve transfection efficiency for gene delivery. Excipients like lactose and sorbitol ensure uniform particle dispersion and effective delivery in nasal vaccines. The adaptability of these combinations highlights PLGA’s versatility for diverse therapeutic applications, from respiratory infections to CNS disorders.

## 4. Factors Affecting Delivery Performance of PLGA-Based Carriers

### 4.1. Impact of Particle Size

Particle size is a critical parameter influencing the delivery performance of PLGA-based carriers. Optimizing particle size allows for improved deposition, cellular uptake, and therapeutic efficacy in pulmonary and intranasal applications, as well as for disease-specific targeting.

#### 4.1.1. Optimal Particle Size for Pulmonary Delivery

For efficient aerosol deposition and deep lung penetration, particle sizes between 1 and 5 µm are widely recognized as optimal. PLGA microparticles, such as sildenafil-loaded microparticles with aerodynamic diameters of 4.74 ± 0.09 µm, have demonstrated enhanced lung deposition and prolonged retention, resulting in effective therapeutic outcomes [59]. Similarly, insulin-loaded PLGA microspheres (4.02 µm) successfully reached alveolar regions, prolonging hypoglycemic effects in animal models [39,81].

Porous particles further enhance pulmonary delivery due to their reduced density and macrophage evasion. Porous sildenafil-loaded PLGA microparticles (6–14 µm) and tadalafil-loaded PLGA microparticles (~10.29 µm) demonstrated deep lung deposition and efficient aerosolized delivery [16,34,53,59]. Such large-porous particles also supported sustained drug release and prolonged lung retention, making them ideal for chronic pulmonary conditions.

#### 4.1.2. Nanoparticles for Pulmonary Applications

PLGA nanoparticles for pulmonary delivery typically range from 100 nm to 1 µm, enabling efficient alveolar deposition and cellular uptake. For example, levofloxacin-loaded nanoparticles (146 nm) achieved high pulmonary deposition and potent antimicrobial activity [24]. Similarly, α1AT-loaded nanoparticles (100 nm to 1 µm) effectively targeted respiratory tract regions for localized therapeutic effects [38].

Porous nanoparticle designs, such as docetaxel-loaded PLGA microparticles (1–5 µm), have further enhanced pulmonary retention by evading macrophage phagocytosis while enabling sustained drug release [27]. These findings underscore the importance of tailoring particle size and porosity to achieve therapeutic benefits in pulmonary conditions.

#### 4.1.3. Particle Size for Intranasal Delivery and Brain Targeting

Small particle sizes (<200 nm) are essential for intranasal drug delivery, allowing for efficient mucosal adhesion, permeation, and subsequent brain targeting. For instance, huperzine A-loaded lactoferrin-modified PLGA nanoparticles (153.2 ± 13.7 nm) successfully delivered drugs from the nasal cavity to the brain for Alzheimer’s therapy [40]. Similarly, RVG29-modified PLGA nanoparticles optimized for nose-to-brain delivery achieved efficient CNS targeting [83].

Chitosan-coated PLGA nanoparticles have further improved nasal delivery by enhancing mucoadhesion and permeation. Gabapentin-loaded nanoparticles (179.4 nm) and curcumin-loaded core/shell nanoparticles (~200 nm) demonstrated efficient nasal tissue adhesion and enhanced bioavailability [62,64]. Such size-optimized formulations highlight the importance of particle size in achieving brain-targeted therapies.

#### 4.1.4. Disease-Specific Particle Size Optimization

Disease-specific particle size optimization is critical for enhancing therapeutic outcomes, as particle size requirements vary depending on the targeted disease. For pulmonary fibrosis, porous PLGA microparticles and nanoparticles have shown improved deposition and retention in fibrotic lungs, with nintedanib-loaded microgels (~12 µm) and thymoquinone-loaded nanoparticles (~20 nm) demonstrating significant therapeutic benefits [33,50]. Similarly, rosmarinic acid-loaded microparticles (~17 µm) effectively targeted fibrotic lung tissues, enabling prolonged drug release [49]. In glioblastoma therapy, intranasal administration of temozolomide-loaded PLGA nanoparticles (<200 nm) achieved efficient brain delivery, overcoming tumor resistance and improving survival outcomes [63]. For tuberculosis, rifampicin-loaded PLGA microparticles (~27.38 µm) ensured retention on the bronchial mucosa, providing sustained drug release tailored to disease-specific needs [53].

Particle size is a fundamental determinant of the performance of PLGA-based carriers in pulmonary and intranasal drug delivery. Optimized particle sizes in the range of 1–5 µm for pulmonary applications and <200 nm for intranasal delivery significantly enhance deposition, retention, and bioavailability. Additionally, porous particles and nanoparticles with tailored aerodynamic properties enable deeper lung penetration and improved biofilm penetration in diseases like cystic fibrosis. Despite these advancements, challenges such as batch-to-batch variability, scalability in particle manufacturing, and the dynamic nature of diseased microenvironments remain barriers to widespread clinical adoption.

### 4.2. Impact of Size Distribution

Size distribution is a critical determinant of the delivery performance of PLGA-based carriers, influencing aerodynamic behavior, drug deposition, and therapeutic outcomes. Uniform size distribution ensures reproducibility, efficient pulmonary or nasal delivery, and enhanced stability [13,18,52,90].

#### 4.2.1. Uniform Size Distribution for Pulmonary Delivery

Achieving uniform size distribution improves aerodynamic performance and lung deposition. Sildenafil-loaded porous PLGA microparticles with narrow size distributions demonstrated enhanced deposition and deep lung retention [59]. Similarly, levofloxacin-loaded PLGA microparticles achieved fine particle fractions (FPFs) suitable for pulmonary administration due to their consistent size distribution [45].

Advanced techniques, such as Flow Focusing^®^ technology, produced uniform tobramycin-loaded PLGA microparticles with reproducible aerodynamic behavior [9]. Similarly, hot-melt extruded sildenafil microparticles displayed consistent geometric size distributions, ensuring predictable pulmonary deposition [18].

#### 4.2.2. Controlled Size Distribution in Porous Particles

Porous PLGA microparticles with controlled size distributions avoid phagocytosis and enable sustained drug release. One-step emulsification produced porous particles with uniform diameters and consistent aerodynamic performance [5]. Electrosprayed porous PLGA microspheres demonstrated homogeneous size distributions optimized for inhalable formulations [10]. Additionally, rifampicin-loaded microparticles modified with L-leucine increased the FPF (43.4%), improving deposition efficiency [54].

#### 4.2.3. Narrow Size Distribution in Nanoparticles

Narrow size distributions in nanoparticle formulations enhance stability, cellular uptake, and therapeutic efficacy. Curcumin-loaded nanoparticles with odorranalectin modification exhibited uniform size distribution, ensuring consistent drug uptake [84]. Similarly, topiramate-loaded PLGA nanoparticles with a polydispersity index (PDI) of 0.202 achieved efficient nasal mucosal permeation [65].

Favipiravir-loaded nanoparticles with a PDI of 0.130 ensured predictable delivery performance [68]. DNase I-loaded nanoparticles were optimized to achieve a narrow size distribution (below 6 µm), enhancing lung deposition [37]. PLGA-lipid hybrid nanoparticles (HNPs) for siRNA delivery also demonstrated narrow size distributions, enabling consistent transfection efficiency [75].

#### 4.2.4. Effect of Size Distribution on Intranasal Delivery

Uniform size distributions are crucial for efficient mucosal uptake and nasal-to-brain drug delivery. PLGA–PEG nanocarriers exhibited homogeneous size distributions, enabling consistent delivery to the brain for Alzheimer’s disease therapy [32]. Huperzine A-loaded nanoparticles with a PDI of 0.229 ± 0.078 provided uniform drug release and therapeutic efficacy [40]. TMZ16e-loaded PLGA nanoparticles for glioblastoma therapy also demonstrated narrow size distributions, enhancing brain targeting [63].

#### 4.2.5. Technological Approaches for Size Control

Various preparation techniques are employed to achieve controlled size distributions. Block copolymer-assisted emulsification produced PEG-PLA/PLGA microparticles with uniform sizes (~2 µm), ideal for pulmonary applications [91]. Spray-drying methods yielded vitamin D3-loaded PLGA microspheres with a PDI of 0.509, ensuring consistent aerodynamic performance [25]. Electrosprayed particles and premix membrane homogenization further enabled reproducible size distributions for pulmonary delivery [15], [2]. Response surface methodology (RSM) was used to optimize PLGA microparticles with controlled size distributions, improving efficiency for pulmonary applications [82].

#### 4.2.6. Polydispersity and Stability

A low PDI is essential for ensuring size uniformity and predictable performance. Alpha-mangostin-loaded PLGA microspheres demonstrated a PDI of 0.95 ± 0.009, confirming uniform size distribution [17]. Ciprofloxacin-loaded nanoparticles achieved narrow distributions (PDI ranging from 0.050 to 1.00), improving penetration through biofilms in cystic fibrosis [31]. Size distribution also impacts stability. Etoposide-loaded PLGA microspheres maintained stable size distributions after freeze-drying [92]. Fluorescent PLGA nanoparticles exhibited monodisperse distributions confirmed by dynamic light scattering (DLS), ensuring consistent delivery potential for pulmonary and intranasal applications [90].

The size distribution of PLGA-based carriers is a critical factor influencing their therapeutic performance in pulmonary and intranasal delivery. Uniform size distributions enhance drug deposition, cellular uptake, and bioavailability while reducing variability in therapeutic outcomes. Techniques like emulsification, spray-drying, and electrospraying have enabled the production of particles with narrow size distributions, but scalability and batch-to-batch consistency remain significant challenges. For nanoparticles, achieving a low polydispersity index (PDI) is vital for stability and predictable drug release, yet this often requires labor-intensive processes. Advanced manufacturing methods and real-time particle monitoring could address these limitations, ensuring consistent size distribution during large-scale production. Furthermore, variability in physiological conditions, such as mucus composition and airflow dynamics, highlights the need for size-controlled formulations tailored to individual patient needs. Continued research into predictive modeling, stabilizing excipients, and scalable production techniques will be essential to fully realize the potential of size-optimized PLGA carriers in clinical applications.

### 4.3. Impact of Particle Aerodynamic Performance

The aerodynamic performance of PLGA-based carriers is crucial for efficient drug delivery in pulmonary applications. Key factors such as aerodynamic diameter, porosity, density, and fine particle fraction (FPF) directly influence drug deposition, retention, and therapeutic efficacy in the lungs. Below is a condensed analysis of the role of aerodynamic performance in optimizing delivery.

#### 4.3.1. Optimal Aerodynamic Diameter for Lung Deposition

Particles with aerodynamic diameters between 1 and 5 μm are ideal for deep lung deposition. For example, levofloxacin-loaded PLGA nanoparticles achieved a mass median aerodynamic diameter (MMAD) of 4.40 μm, facilitating efficient lung deposition [24]. Similarly, sildenafil-loaded porous microparticles demonstrated MMAD values of 6–13 μm, making them suitable for deep lung delivery [18]. Nafarelin acetate-loaded PLGA microspheres achieved a respirable fraction (RF) exceeding 50%, ensuring optimal deposition in the alveolar region [81].

Spray-dried felodipine-loaded PLGA composites exhibited MMAD values ranging from 1.29 to 12.0 μm, allowing targeted deposition in different lung regions [86]. Additionally, PGE1-HPβCD microparticles achieved aerodynamic diameters in the inhalable range of 1–5 μm, despite their larger particle sizes, enabling efficient pulmonary delivery [60].

#### 4.3.2. Enhanced Aerodynamics Through Porosity

Porous PLGA particles offer superior aerodynamic properties due to their reduced density and increased surface area. Highly porous PLGA microparticles achieved an MMAD of 4.0 ± 1.2 μm and an FPF of 32.0 ± 9.1%, enabling efficient lung deposition [34]. Similarly, large-porous deslorelin-loaded PLGA particles exhibited enhanced aerodynamic performance, prolonging lung retention and enabling systemic delivery [19].

For multi-drug systems, docetaxel and celecoxib-loaded porous PLGA microparticles displayed optimized aerodynamic properties for effective pulmonary deposition [27]. The addition of L-leucine improved the FPF of rifampicin-loaded PLGA microparticles by 6.9 times (43.4%), enhancing aerosolization and macrophage phagocytosis [54].

#### 4.3.3. Efficient Aerosolization for Pulmonary Applications

Efficient aerosolization is key to delivering PLGA-based carriers deep into the respiratory tract. Electrosprayed porous microspheres demonstrated ideal aerodynamic diameters for effective lung deposition both in vitro and in vivo [10]. Spray-dried PLGA microparticles embedded in lactose also displayed favorable flow properties, improving aerosolization for pulmonary delivery [23].

Insulin-loaded PLGA microspheres, formulated with sieved mannitol carriers, achieved the highest FPF (18.3 ± 1.65%), enhancing dispersibility and deep lung deposition [35]. Nebulized DNase I-loaded PLGA nanoparticles achieved a respirable fraction of 71.3% (particles <6 μm), optimizing inhalation therapy [37]. Similarly, simvastatin-loaded PLGA nanoparticles demonstrated effective nebulization and therapeutic outcomes in pulmonary fibrosis treatment [51].

#### 4.3.4. Targeted Lung Retention and Cellular Uptake

Aerodynamic properties play a significant role in ensuring localized delivery and retention in lung tissues. Tadalafil-loaded PLGA microspheres, with an aerodynamic diameter of 3.92 μm, ensured efficient lung retention and targeted drug deposition [16]. Rifapentine-linezolid-loaded PLGA microspheres demonstrated prolonged bronchial mucosa retention for up to 20 days, supporting sustained therapeutic outcomes [53].

Anionic PLGA nanoparticles showed superior retention in lung lobules and targeted macrophage accumulation, making them suitable for pulmonary immunomodulatory therapies [4]. DNA-loaded PLGA microcapsules with a geometric mean aerodynamic diameter (GMAD) of 3.43 μm ensured effective deposition in the respiratory tract and sustained gene release [77].

#### 4.3.5. Role of Fine Particle Fraction (FPF) in Delivery Efficiency

The fine particle fraction (FPF) represents the proportion of particles capable of reaching the alveolar region and is critical for efficient pulmonary delivery. Ethionamide-loaded PLGA nanoparticles in a dry powder inhaler achieved an aerodynamic particle size of 1.79 μm, ensuring high FPF and deep lung deposition [30]. Ciprofloxacin-loaded PLGA nanoparticles also demonstrated high aerodynamic performance, enabling penetration through biofilms in cystic fibrosis lungs [31].

Levofloxacin-loaded PLGA microparticles achieved an FPF of 50.99%, ensuring efficient deposition in the alveolar region [45]. Highly porous PLGA microparticles demonstrated an FPF of 32.0 ± 9.1%, further highlighting their suitability for inhalation therapies [34].

#### 4.3.6. Improved Aerodynamics via Particle Engineering

Particle engineering techniques have been instrumental in optimizing aerodynamic properties for pulmonary drug delivery. Response surface methodology was used to produce PLGA microparticles with aerodynamic sizes between 1 and 5 μm, ideal for inhalation therapies [82]. The addition of L-leucine enhanced the aerodynamic behavior of rifampicin-loaded microparticles by reducing particle aggregation and increasing dispersibility [54]. Surface modifications further influence aerodynamic performance. Chitosan-coated PLGA microspheres enhanced nasal retention and reduced clearance, supporting improved mucosal immune responses [69]. PLGA-lipid hybrid nanoparticles showed improved mucus penetration and stability in bronchoalveolar lavage fluid (BALF), making them effective for siRNA delivery [75].

The aerodynamic performance of PLGA-based carriers plays a central role in achieving efficient pulmonary drug delivery. By optimizing parameters such as aerodynamic diameter, porosity, and fine particle fraction (FPF), these formulations can achieve enhanced lung deposition, prolonged retention, and targeted delivery. Techniques like spray-drying, electrospraying, and surface modifications have improved particle dispersibility and aerodynamic properties, particularly for porous formulations. However, achieving consistent aerodynamic performance across diverse patient populations and scaling these methods for industrial production remain major challenges. Further research is needed to address variability caused by environmental conditions, patient-specific factors, and storage stability. Integrating computational modeling, real-time monitoring, and machine learning could accelerate the optimization of aerodynamic parameters, enhancing reproducibility and efficiency.

### 4.4. Impact of Surface Charge

Surface charge significantly influences the delivery performance of PLGA-based carriers by affecting colloidal stability, cellular uptake, mucoadhesion, immune response, and drug targeting [20,25,40]. Modulating surface charge through coatings or chemical modifications optimizes the functionality of PLGA nanoparticles and microparticles for pulmonary and intranasal drug delivery.

#### 4.4.1. Colloidal Stability and Surface Charge

Surface charge contributes to the colloidal stability of PLGA formulations in biological environments. Both positively and negatively charged nanoparticles demonstrate stability in biological media and during cryo-storage, ensuring reproducible performance. Favipiravir-loaded PLGA nanoparticles with a zeta potential of −17.1 mV showed enhanced stability for nasal delivery [68]. Similarly, mannosylated PLGA nanoparticles with a negative zeta potential of −33.1 mV exhibited excellent colloidal stability [66].

Surface coatings, such as PEG, further enhance stability by modifying zeta potential. PEG-PLA/PLGA microparticles showed increased zeta potential after PEG modification, ensuring improved dispersion and functionality [91]. Similarly, topiramate-loaded PLGA nanoparticles with a zeta potential of −12.7 mV maintained stability for nasal drug delivery [65].

#### 4.4.2. Surface Charge and Cellular Uptake

The interaction of PLGA carriers with cells is influenced by their surface charge. Negatively charged nanoparticles often show faster and higher uptake rates. For instance, anionic PLGA nanoparticles exhibited a 10-fold increase in uptake by macrophages and a 2.5-fold increase by alveolar epithelial cells compared to cationic particles, making them effective for targeting lung macrophages [4].

Conversely, positive surface charges improve uptake in specific applications. Lactoferrin-coated PEG-PLGA nanoparticles achieved enhanced brain delivery due to their positive charge from lactoferrin modification [12]. Similarly, chitosan-coated PLGA nanoparticles demonstrated enhanced cellular uptake and mucoadhesion in nasal delivery systems [21,22].

#### 4.4.3. Mucoadhesion and Nasal Retention

Positively charged PLGA carriers enhance mucoadhesion and retention on mucosal surfaces, which is critical for nasal and pulmonary delivery. Chitosan-coated PLGA nanoparticles with zeta potentials ranging from +33.47 mV to +50.13 mV exhibited prolonged nasal retention and superior mucosal adhesion compared to uncoated particles [29]. Hepatitis B antigen-loaded PLGA microspheres with a trimethylchitosan (TMC) coating achieved a highly positive zeta potential of +27.2 mV, enhancing nasal retention and immune response [69].

In pulmonary drug delivery, positively charged LMWC-coated PLGA nanoparticles improved adhesion to lung tissues, facilitating efficient drug delivery and retention [28,29].

#### 4.4.4. Impact of Charge on Immune Response and Drug Targeting

Surface charges modulate immune responses and tissue targeting. TMC-coated PLGA nanoparticles for nasal vaccination enhanced mucosal adhesion and antigen delivery, stimulating dendritic cell maturation and improving immune responses [76]. PLGA–PEI nanoparticles with a zeta potential above +30 mV demonstrated improved DNA adsorption and pulmonary immune activation, making them ideal for DNA vaccine delivery [74].

In glioblastoma therapy, RGD-conjugated PLGA nanoparticles utilized surface charge modifications to enhance tumor targeting and cellular uptake [44]. For Alzheimer’s therapy, lactoferrin-TMC-coated PLGA nanoparticles with a zeta potential of +35.6 ± 5.2 mV ensured effective nasal-to-brain delivery, improving CNS drug targeting [40].

#### 4.4.5. Charge Modulation for Enhanced Delivery

Modifying surface charge optimizes drug delivery by improving penetration, retention, and targeting. PLGA-lipid hybrid nanoparticles with tunable surface charges enabled efficient mucus penetration and cellular uptake, making them suitable for siRNA delivery [75]. Porous sildenafil citrate-loaded PLGA microparticles modified with polyethyleneimine (PEI) exhibited charge properties conducive to deep lung deposition and sustained drug release [59].

In some cases, neutral or slightly negative charges are beneficial. Aerosolized microgels with nintedanib-PLGA nanoparticles avoided phagocytosis by alveolar macrophages due to their optimized neutral surface properties [33]. Additionally, PEGylation of PLGA nanoparticles reduced mucin interactions, enhancing mucosal penetration and retention [32].

Surface charge is a critical determinant of PLGA nanoparticle performance, affecting colloidal stability, cellular uptake, mucoadhesion, immune modulation, and drug targeting. Optimizing surface charge through precise chemical modifications and coatings ensures improved delivery outcomes while minimizing adverse effects like immune activation or toxicity. Challenges such as achieving long-term stability, maintaining biocompatibility, and overcoming patient-specific variability require continued research. Future advancements should focus on scalable charge modulation techniques and adaptive designs for consistent performance across diverse applications.

### 4.5. Impact of Particle Porosity

Porosity plays a critical role in enhancing the performance of PLGA-based carriers by influencing aerodynamic properties, drug retention, sustained release, and tissue targeting. The presence of pores in particles reduces density, improves drug loading, and optimizes therapeutic efficacy in pulmonary and intranasal drug delivery systems.

#### 4.5.1. Aerodynamic Performance and Pulmonary Deposition

Porous PLGA particles improve aerodynamic properties, enabling efficient pulmonary deposition. Low-density particles, such as those with tapped densities as low as 0.04 g/cm^3^, demonstrated superior aerodynamic behavior and enhanced lung delivery [5]. For instance, large-porous deslorelin-loaded PLGA particles with a density of 0.082 g/cm^3^ exhibited prolonged retention and systemic delivery [19]. Similarly, sildenafil-loaded porous PLGA microparticles engineered with ammonium bicarbonate as a porogen achieved low tapped densities (0.100 ± 0.002 g/cm^3^), optimizing them for deep lung deposition [59].

Internal pore structures further enhance aerodynamic properties. Porous PLGA particles with spontaneous emulsification-based pores exhibited better aerosolization due to reduced density and increased surface area [59,60]. Dimpled PLGA microcapsules with a density of 0.24 g/cm^3^ showed excellent aerosolization efficiency, making them suitable for pulmonary applications [77].

#### 4.5.2. Prolonged Drug Retention and Sustained Release

Porous structures enable sustained drug release, maintaining therapeutic concentrations over extended periods. Artesunate-loaded porous PLGA microspheres with highly porous morphologies supported prolonged release and efficient lung cell uptake [43]. Ciprofloxacin-loaded porous microspheres also demonstrated slow release, enabling penetration through CF biofilms and extending antibacterial activity [31].

Dual drug-loaded porous PLGA microspheres containing doxorubicin and paclitaxel were designed with optimized porosity for controlled release and effective lung targeting [26]. Similarly, insulin-loaded porous nanospheres facilitated extended therapeutic action by sustaining drug retention in the pulmonary region [46].

Porous microgels encapsulating nintedanib-PLGA nanoparticles provided prolonged antifibrotic effects by extending drug release and reducing fibrotic progression in lung tissues [33]. Additionally, porous rifampicin-linezolid PLGA microspheres with circular concave morphologies ensured sustained drug retention on bronchial mucosa [53].

#### 4.5.3. Drug Loading and Tissue Targeting

Porosity enhances drug loading efficiency and supports targeted tissue delivery. Hydrophilic polymers such as alginate and chitosan modified the porosity of PLGA nanoparticles, improving deposition in specific airway regions [23]. Galantamine adsorption onto hierarchical porous carbon (HPC) increased drug loading capacity while ensuring controlled release for Alzheimer’s treatment [93].

Porous PLGA carriers have also been developed for vaccine delivery. For instance, PLGA microspheres loaded with malaria antigens enabled prolonged immune responses through sustained antigen release [88]. Similarly, porous PLGA nanoparticles encapsulating BPI3V antigens enhanced mucosal immunity by supporting controlled antigen release [70].

#### 4.5.4. Porogen-Assisted Porosity Engineering

Porogen-based methods are widely used to enhance porosity in PLGA particles, improving drug release and aerodynamic properties. Sodium bicarbonate as a porogen in sildenafil-loaded microparticles introduced internal pores, creating brittle particles with favorable aerodynamic properties [18]. Similarly, ammonium bicarbonate was used to generate high porosity in PLGA microparticles, facilitating deep pulmonary deposition [34].

Supercritical fluid technology was employed to produce celecoxib-loaded porous PLGA microparticles, where porosity enhanced drug delivery and lung retention [8]. Porogen-assisted techniques provide a versatile approach to tailoring porosity for various therapeutic applications.

#### 4.5.5. Enhanced Mucosal and Cellular Uptake

Porous particles exhibit superior interaction with biological tissues, enabling drug delivery across mucosal barriers and improving cellular uptake. Chitosan-modified porous PLGA nanoparticles demonstrated enhanced mucoadhesion and prolonged retention on mucosal surfaces [21,22]. Additionally, core–shell PLGA-lipid hybrid nanoparticles with a porous lipid core achieved controlled siRNA release and effective cellular targeting, further highlighting the utility of porosity in delivery performance [75].

Porosity is a key parameter in PLGA-based drug delivery systems, significantly enhancing aerodynamic properties, sustained release, drug loading, and tissue targeting. Porous structures improve therapeutic efficacy by increasing deposition efficiency and prolonging drug action, especially in pulmonary and mucosal applications. However, achieving consistent porosity during production remains a significant challenge, as variations can affect performance, stability, and scalability. Future research should focus on advanced engineering techniques to optimize pore architecture while addressing challenges such as drug diffusion into pores, structural integrity, and regulatory compliance.

### 4.6. Impact of Particle Morphology

Particle morphology significantly influences the performance of PLGA-based drug carriers, particularly in pulmonary and intranasal delivery systems. The shape, surface features, and structural uniformity of particles impact aerodynamic behavior, drug release, and interaction with biological tissues, ultimately enhancing therapeutic efficacy.

#### 4.6.1. Spherical and Smooth Morphology for Stability and Delivery Efficiency

Spherical particles enhance stability, improve aerodynamic properties, and facilitate efficient drug delivery. PAMAM-modified PLGA nanoparticles with smooth, spherical surfaces enabled efficient mucosal penetration for nasal delivery [11]. Similarly, insulin-cyclodextrin complex-loaded PLGA microspheres exhibited smooth surfaces, improving dispersibility and minimizing aggregation for pulmonary delivery [36].

Favipiravir-loaded PLGA nanoparticles displayed spherical and smooth surfaces, contributing to stability and optimized drug delivery [68]. α1AT-loaded PLGA nanoparticles, with their spherical morphology, ensured uniform drug distribution and efficient aerodynamic performance in pulmonary applications [38]. Spherical morphologies also reduced device adhesion, as demonstrated by nafarelin acetate-loaded PLGA microspheres [81].

#### 4.6.2. Rough and Textured Morphologies for Aerodynamic Efficiency

Rough and porous textures enhance aerodynamic performance, drug retention, and controlled release. Electrosprayed PLGA microspheres with rough, porous surfaces proved ideal for pulmonary drug delivery due to improved deposition efficiency [10]. Spray-dried PLGA microparticles with rough textures and internal porosity enhanced aerodynamic behavior and lung deposition [15].

Microparticles with smooth yet porous surfaces, such as PEI-modified PLGA carriers, combined effective drug loading, controlled release, and improved lung retention [58].

#### 4.6.3. Uniformity and Homogeneity in Morphology

Homogeneous and uniform morphologies ensure consistent aerodynamic performance, predictable drug release, and reproducible therapeutic effects. Tobramycin-loaded PLGA microparticles displayed uniform morphology, supporting reproducible aerosolization [9]. Similarly, vitamin D3-loaded PLGA microspheres with smooth, uniform surfaces facilitated sustained nasal permeation [25].

Uniform spherical morphology is particularly advantageous in vaccine delivery. CNA-loaded PLGA nanoparticles for nasal vaccine applications displayed consistent spherical morphology, which enhanced immunogenicity and nasal retention [71]. Aβ1–15-loaded PLGA microparticles with uniform morphology ensured efficient delivery of antigens for Alzheimer’s immunotherapy [89]. Rifampicin-loaded PLGA microspheres also retained stable spherical morphology during hydrolytic degradation, ensuring consistent performance [55].

#### 4.6.4. Surface Features for Targeted Delivery

Specific surface features of PLGA particles enhance interaction with biological tissues, supporting targeted delivery. Trimethylchitosan-modified PLGA nanoparticles exhibited smooth, spherical surfaces, enhancing mucoadhesion and nasal retention [94]. Rifampicin-loaded PLGA particles modified with L-leucine achieved a non-spherical shape, improving aerodynamic performance for pulmonary delivery [54].

PEG-PLA/PLGA microparticles with dimpled spherical morphology increased surface area and facilitated drug release, optimizing them for inhalation therapies [91]. Similarly, TMZ16e-loaded PLGA nanoparticles with anti-EphA3 surface modifications demonstrated uniform morphology and improved targeting of glioblastomas [63].

Particle morphology is a pivotal factor in determining the performance of PLGA-based drug delivery systems. Spherical and smooth morphologies enhance stability and dispersibility, while porous and textured designs improve aerodynamic properties, sustained release, and drug retention. However, challenges such as achieving uniformity, structural stability, and scalable production persist. Advanced fabrication methods like microfluidics, spray drying, and electrospraying, combined with real-time monitoring tools, can help overcome these limitations. Future efforts should focus on tailoring particle morphology to specific therapeutic needs while ensuring reproducibility and stability during manufacturing and storage.

### 4.7. Impact of Drug Loading and Encapsulation Efficiency

Drug loading and encapsulation efficiency are key factors influencing the performance of PLGA-based drug delivery systems. These parameters impact drug payload, release profiles, stability, and therapeutic efficacy. Below is a condensed discussion of their critical roles, supported by precise examples.

#### 4.7.1. High Encapsulation Efficiency for Enhanced Drug Retention

High encapsulation efficiency maximizes drug retention within PLGA carriers, enabling prolonged drug release and improved therapeutic outcomes. For instance, amiodarone-loaded PLGA nanoparticles achieved 88% encapsulation efficiency, ensuring sustained pulmonary delivery [7]. Similarly, rhIL-2-loaded PLGA microparticles demonstrated 99.22% encapsulation efficiency while maintaining protein integrity for effective pulmonary therapy [39]. Tadalafil-loaded PLGA microspheres achieved 81.68% encapsulation efficiency with 8.52% drug loading, supporting controlled drug release [16].

In vaccine delivery, PLGA nanoparticles encapsulating *Klebsiella pneumoniae* capsular antigen provided stable encapsulation for effective pulmonary immune responses [95]. PLGA–PEI nanoparticles also exhibited >99% DNA loading, ensuring sustained pulmonary immune responses for DNA vaccination [74]. For siRNA delivery, PLGA-lipid hybrid nanoparticles achieved high encapsulation efficiencies, enabling efficient gene silencing in lung cancer models [75].

#### 4.7.2. Optimized Drug Loading for Sustained Release

Optimizing drug loading ensures sustained release and therapeutic availability. Etoposide-loaded PLGA microspheres achieved 7.7 ± 0.3% drug loading and 84.2 ± 2.9% encapsulation efficiency, enabling extended pulmonary drug delivery [81]. Sildenafil-loaded microparticles exhibited drug loading between 17 and 33% w/*w*, with 77–89% encapsulation efficiency, providing a sustained release for pulmonary applications [18].

Rosmarinic acid-loaded PLGA microspheres achieved 24.97 ± 0.97% drug loading, enabling localized release for pulmonary fibrosis treatment [49]. Insulin-loaded PLGA microspheres demonstrated 14.76 ± 1.1% drug loading, supporting controlled release for prolonged hypoglycemic effects [36]. In Alzheimer’s therapy, huperzine A-loaded Lf-TMC nanoparticles exhibited 73.8 ± 5.7% entrapment efficiency for effective brain targeting [40].

#### 4.7.3. Role of Surface Modifications in Enhancing Encapsulation

Surface modifications improve encapsulation efficiency and drug release properties. Chitosan-coated PLGA nanoparticles significantly enhanced the encapsulation efficiency of ropinirole hydrochloride for nasal-to-brain delivery [13]. Similarly, LMWC-coated PLGA nanoparticles demonstrated controlled drug encapsulation and prolonged release based on chitosan concentration [29].

Tobramycin-loaded PLGA nanoparticles encapsulated with alginate as a stabilizer achieved improved encapsulation efficiency, facilitating sustained pulmonary drug release [23]. For curcumin delivery, odorranalectin-modified nanoparticles enhanced drug loading and bioavailability compared to unmodified formulations [84].

#### 4.7.4. High Encapsulation Efficiency for Combination Therapies

PLGA carriers are particularly effective for dual-drug therapies, where encapsulation efficiency influences synergistic therapeutic effects. Dual-drug-loaded porous PLGA microspheres containing docetaxel and celecoxib demonstrated high drug encapsulation efficiency, enabling prolonged release and enhanced anticancer effects [27]. Similarly, porous microparticles encapsulating doxorubicin and paclitaxel achieved optimized encapsulation for lung targeting and sustained release [26].

Nib-PLGA-DPPs loaded with nintedanib achieved 15.7% drug loading, enabling effective pulmonary fibrosis treatment [57]. Rifapentine-linezolid-loaded PLGA microspheres achieved drug loading of 18.51% and 8.42%, respectively, with encapsulation efficiencies of 55.53% and 16.87%, ensuring retention on bronchial mucosa and sustained delivery [53].

#### 4.7.5. Encapsulation for Enhanced Stability and Biological Activity

High encapsulation efficiency preserves biological activity, ensuring effective therapeutic delivery. DNase I-loaded PLGA nanoparticles retained 76% enzymatic activity post-encapsulation, enabling efficient lung deposition [37]. Similarly, α1AT-loaded PLGA nanoparticles maintained high encapsulation efficiency and protein stability, achieving effective respiratory delivery [38].

TRAIL-neutralizing antibody-loaded PLGA nanoparticles exhibited ~99% encapsulation efficiency, retaining therapeutic efficacy for Alzheimer’s disease [67]. For vaccine applications, chitosan-coated PLGA microspheres encapsulating hepatitis B antigen achieved 80 ± 5% antigen loading, enabling prolonged nasal retention and robust immune responses [69].

#### 4.7.6. Impact of Formulation Techniques on Encapsulation

Formulation techniques significantly impact drug loading and encapsulation efficiency. Modified double emulsion methods increased the drug loading of levofloxacin-loaded PLGA microspheres for sustained pulmonary therapy [2]. Spray-dried formulations, such as curcumin-loaded PLGA nanoparticles, improved therapeutic efficacy due to optimized encapsulation during production [15].

Porous particles also enhance encapsulation efficiency. Self-encapsulating PLGA microspheres achieved 0.60 ± 0.05% ovalbumin loading, enabling prolonged vaccine release [73]. Ammonium bicarbonate-assisted porous PLGA microparticles encapsulated lysozyme and doxorubicin with ~100% efficiency, demonstrating the potential for pulmonary delivery [34].

Drug loading and encapsulation efficiency are essential parameters for optimizing PLGA-based drug delivery systems. High encapsulation efficiency improves therapeutic outcomes through prolonged drug release and retention, while optimized drug loading ensures sustained availability. Surface modifications and advanced formulation techniques further enhance these parameters but require careful balancing to avoid compromising particle stability, biocompatibility, or scalability. To address challenges such as variability in batch production and the encapsulation of sensitive biologics, future efforts should focus on integrating real-time monitoring tools and developing innovative stabilization strategies tailored to specific therapeutic applications.

### 4.8. Impact of Drug Release

The drug release profile is a key determinant of the therapeutic efficacy and delivery performance of PLGA-based carriers. Controlled and sustained drug release from these carriers ensures prolonged therapeutic effects, enhanced drug targeting, reduced systemic toxicity, and improved patient compliance. Below is a condensed discussion on the impact of drug release profiles in various applications.

#### 4.8.1. Sustained Release for Prolonged Therapeutic Effects

PLGA carriers are well-known for their ability to sustain drug release over extended periods, ensuring continuous therapeutic effects. Etoposide-loaded PLGA microspheres released the drug over 20 days, following the Ritger-Peppas model, supporting prolonged therapeutic effects for pulmonary applications [92]. Tadalafil-loaded PLGA microspheres released 84.06% of the drug over 10 days, highlighting their suitability for pulmonary hypertension treatment [16]. Similarly, sildenafil-loaded PLGA microparticles demonstrated 70% cumulative drug release over seven days [18].

Porous deslorelin-loaded PLGA microparticles sustained drug release over seven days, achieving higher plasma concentrations than smaller, non-porous particles [19]. Artesunate-loaded porous microspheres also enabled drug release over eight days, improving outcomes in lung cancer therapy [43]. For antifibrotic applications, aerosolized microgels encapsulating nintedanib-PLGA nanoparticles provided prolonged drug release, reducing fibrosis progression in lung tissue [33].

#### 4.8.2. Prolonged Release for Targeted Pulmonary Delivery

In targeted pulmonary delivery, sustained drug release is essential for localized and prolonged effects. Levofloxacin-loaded PLGA nanoparticles sustained drug release for 120 h, ensuring consistent antibiotic levels in the lungs [24]. Ciprofloxacin-loaded PLGA nanoparticles also maintained therapeutic concentrations for extended periods, allowing penetration through mucus layers in cystic fibrosis lungs [31].

Insulin-cyclodextrin complex-loaded PLGA microspheres provided controlled insulin release, resulting in extended hypoglycemic effects [36]. Highly porous PLGA microparticles encapsulating lysozyme and doxorubicin achieved 52% cumulative drug release over four days, supporting local pulmonary drug delivery [34].

#### 4.8.3. Controlled Drug Release for Brain-Targeted Delivery

Sustained drug release from PLGA carriers enhances nose-to-brain delivery by increasing brain residence time. Oxcarbazepine-loaded PLGA nanoparticles released 53.5% of the drug over 24 h, enabling once-daily administration for seizure management [96]. Huperzine A-loaded Lf-TMC PLGA nanoparticles sustained drug release for 48 h, ensuring effective Alzheimer’s treatment [40]. RGD-conjugated PLGA nanoparticles allowed extended doxorubicin release, achieving glioblastoma-specific targeting and prolonged therapeutic effects [44].

#### 4.8.4. Dual-Drug and Multi-Drug Release Profiles

PLGA carriers are ideal for combination therapies, as they enable the controlled release of multiple agents for synergistic effects. Doxorubicin- and paclitaxel-loaded porous PLGA microspheres provided sustained dual-drug release, optimizing combination therapy for lung cancer [26]. Rifapentine-linezolid-loaded microspheres exhibited burst release followed by sustained drug release over 14 days, improving tuberculosis treatment with cumulative releases of 27.61% (rifapentine) and 51.01% (linezolid) [53]. Similarly, BoR-Cur/Cis nanoparticles prolonged dual-drug release, improving survival in pediatric brainstem glioma models [61].

#### 4.8.5. Biphasic and Controlled Release Profiles

Many PLGA carriers exhibit biphasic drug release, combining an initial burst release with a sustained phase. Ethionamide-loaded PLGA nanoparticles demonstrated a biphasic release profile, with a rapid initial release followed by zero-order sustained release (95.17 ± 3.59% release over 24 h) [30]. DNase I-loaded PLGA nanoparticles achieved prolonged enzymatic activity, supporting efficient mucus clearance in pulmonary therapy [37]. Similarly, rhIL-2-loaded PLGA microparticles showed a biphasic release pattern, enabling extended therapeutic effects over multiple days [39].

#### 4.8.6. Effect of Surface Modifications on Drug Release

Surface modifications significantly influence drug release profiles. Chitosan-coated PLGA nanoparticles released ropinirole hydrochloride completely within 24 h in nasal delivery, supporting efficient nose-to-brain therapy [13]. LMWC-coated PLGA nanoparticles sustained tobramycin release over two days, with slower release rates observed at higher coating concentrations [29].

In nasal vaccine delivery, chitosan-coated PLGA microspheres enabled controlled antigen release, prolonging immune responses [69]. Similarly, BPI3V antigen-loaded PLGA nanoparticles sustained antigen release, resulting in long-term mucosal IgA responses [70].

#### 4.8.7. Sustained Release for Enhanced Mucosal and Systemic Responses

Prolonged drug or antigen release enhances both mucosal and systemic therapeutic effects. Ovalbumin-loaded PLGA microspheres demonstrated sustained antigen release over 49 days, retaining immunogenicity for nasal vaccine applications [73]. Hepatitis B antigen-loaded PLGA microspheres exhibited controlled release, enhancing nasal retention and immune responses [87]. Favipiravir-loaded PLGA nanoparticles achieved 24 h sustained release, which extended to 32 h in a thermosensitive gel, improving therapeutic outcomes [68].

#### 4.8.8. Prolonged Drug Release Enhancing Pulmonary Retention

Porous structures in PLGA carriers are particularly effective for sustaining drug release and improving pulmonary retention. Rosmarinic acid-loaded PLGA microspheres sustained drug release for seven days, improving efficacy for radiation-induced pulmonary fibrosis [49]. Sildenafil-loaded porous microparticles sustained drug release over 72 h, ensuring targeted pulmonary delivery [59]. Similarly, aviptadil-loaded PLGA microparticles achieved 57% drug release over seven days in simulated lung fluid, supporting treatment for pulmonary hypertension [97].

The drug release profile is a critical determinant of the success of PLGA-based delivery systems, enabling sustained therapeutic effects, localized targeting, and improved patient compliance. While prolonged release profiles and biphasic kinetics are advantageous in extending efficacy, challenges such as polymer degradation variability, burst release, and interpatient differences in physiology must be addressed. Future advancements should focus on integrating computational modeling, surface modifications, and advanced manufacturing methods to achieve more precise and reproducible drug release kinetics, tailored to specific clinical needs.

Table 3 describes the key physicochemical properties of PLGA formulations, including sustained release, particle size, encapsulation efficiency, and surface modifications. It demonstrates how these properties enhance drug delivery performance. PLGA’s controlled release capabilities extend therapeutic effects while reducing dosing frequency. Tailored particle sizes, from nanoscale to microscale, address specific delivery needs such as deep lung deposition or brain targeting. Surface modifications (e.g., cationic/anionic charges) improve mucoadhesion and cellular uptake. Porous structures avoid macrophage uptake, enabling prolonged retention. High encapsulation efficiency and compatibility with hydrophilic and hydrophobic drugs ensure versatility. These properties are critical in developing advanced formulations for targeted, sustained, and efficient drug delivery.

## 5. Techniques for Designing Tailored PLGA Drug Delivery Systems

### 5.1. Emulsion Solvent Evaporation for High Encapsulation Efficiency

Emulsion solvent evaporation is a widely employed technique for preparing PLGA-based formulations in intranasal and pulmonary drug delivery. This method is particularly effective for encapsulating hydrophilic drugs, achieving high encapsulation efficiency and precise control over particle size—critical factors for consistent and controlled drug release [2,36,46]. Stable nanoparticles and microparticles produced by this method are ideal for delivering therapeutics like prostaglandin E1 and deslorelin, ensuring steady and extended release for optimal therapeutic effectiveness [19,58]. Additionally, this method supports surface modifications, such as mannosylation, which enhance targeting capabilities for brain-specific delivery via the nasal route [66].

### 5.2. Nanoprecipitation for Targeted CNS Delivery

**Figure 3:**
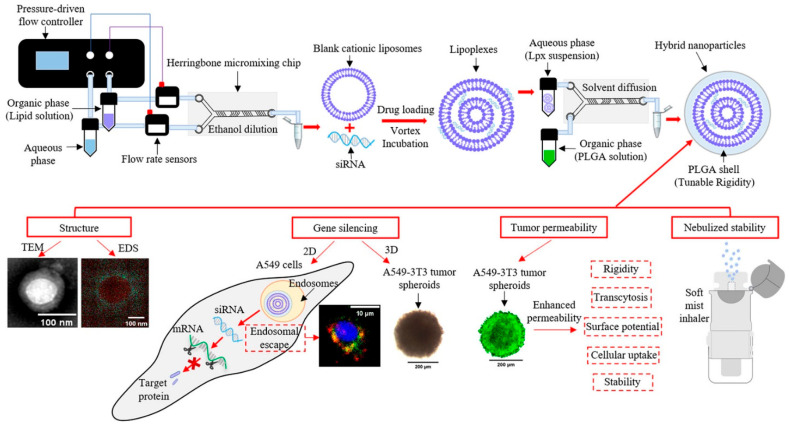
Schematic representation for microfluidic preparation of cationic liposomes (CLs), lipoplexes (Lpx), and hybrid nanoparticles (HNPs) via the herringbone micromixing chip. This includes gene silencing in both 2D- and 3D-cultured cells and tumor permeability in A549-3T3 tumor spheroids for enhanced permeability and stability. Adopted with permission [75].

Nanoprecipitation is another key technique, producing small, stable nanoparticles with uniform size distribution and high encapsulation efficiency. These properties are essential for targeting delicate regions like the CNS. Drugs such as rotigotine and docetaxel benefit from this approach, with precise particle sizes enabling controlled release and targeted brain delivery [11,12,32]. For DNA-based therapies and siRNA formulations, nanoprecipitation ensures stability and effective encapsulation, which are crucial for consistent gene delivery and therapeutic efficacy in respiratory and nasal applications (Figure 3) [75]. The technique’s ability to create small, uniform particles with specific surface properties enhances both bioavailability and uptake in targeted therapies.

### 5.3. Spray-Drying for Pulmonary Drug Delivery

Spray-drying is commonly used to prepare PLGA formulations for pulmonary drug delivery, offering precise control over particle size and aerodynamic properties. This technique is particularly effective for dry powder formulations, enhancing aerosolization and enabling deep lung deposition. Antibiotics like rifampicin and linezolid leverage this method to achieve uniform particle distribution, critical for treating respiratory infections [53]. Spray-drying is also valuable for multi-drug formulations, such as combinations of antibiotics, mucolytics, and anti-inflammatory agents, ensuring consistent distribution of each component within the particles [15,35]. Incorporating mannitol as a carrier further optimizes aerosol performance, making spray-drying adaptable for various respiratory treatments [35].

### 5.4. Freeze-Drying for Stability and Long-Term Storage

Freeze-drying, or lyophilization, enhances stability and enables long-term storage of PLGA formulations. This method is crucial for sensitive biologics, preserving structural integrity and therapeutic potency over extended periods. It is particularly effective for formulations like tadalafil-loaded microspheres, making them suitable for nose-to-brain drug delivery applications where storage stability is critical [16,94]. In vaccine development, freeze-drying helps maintain the efficacy of malaria and hepatitis B vaccines, ensuring their immunogenicity remains intact through prolonged storage [69,88,96].

### 5.5. Advanced Techniques: Electrospraying and Microfluidics

Advanced methods like electrospraying and microfluidics provide precise control over particle characteristics. Electrospraying is highly effective for creating porous microspheres that enhance lung deposition and facilitate sustained drug release—key attributes for treating conditions like lung cancer. Oridonin-loaded PLGA particles prepared via electrospraying achieve controlled release and effective lung deposition [10]. Microfluidics, on the other hand, enables the development of PLGA-lipid core–shell nanoparticles, offering precise control over particle morphology. This technique is particularly beneficial for inhaled siRNA therapies, where specific particle sizes and mucus-penetrating properties are critical for high transfection efficiency [75]. Both methods allow fine-tuning of particle characteristics, enhancing bioavailability and targeted delivery.

### 5.6. Surface Modifications for Enhanced Bioavailability

Surface modifications further improve the bioavailability and targeting capabilities of PLGA particles. Mannosylated ligands, for instance, enhance uptake by macrophages, supporting nasal formulations designed for CNS delivery [3,66]. Ionic complexation techniques, such as TMC-coated PLGA particles, improve mucoadhesion, extend nasal residence time, and promote dendritic cell activation. These enhancements are particularly useful in immunotherapies, boosting vaccine efficacy by enhancing immune responses against respiratory pathogens [76]. Surface modification ensures therapeutic agents reach their intended targets effectively and remain bioavailable for the necessary duration.

### 5.7. Emerging Techniques: Flow Focusing^®^ and Supercritical Fluid Processing

Innovative techniques such as Flow Focusing^®^ technology and supercritical fluid processing refine PLGA particle properties for specific applications. Flow Focusing^®^ technology offers precise control over particle size, making it ideal for creating particles with sustained release profiles for treating chronic respiratory infections [9]. Supercritical fluid processing produces large, porous microparticles, such as celecoxib-loaded formulations, which minimize toxicity while achieving prolonged therapeutic effects [8]. Top-down particle engineering creates unique discoidal shapes that improve lung deposition and resist macrophage uptake, enhancing efficacy in chronic pulmonary conditions like fibrosis [57].

### 5.8. Optimization Tools for Tailored PLGA Formulations

Sophisticated optimization techniques, including Box–Behnken design and factorial design, further enhance PLGA formulations for specific delivery objectives. These methods optimize parameters like particle size, zeta potential, and drug loading, which directly impact bioavailability, release profiles, and delivery efficiency. For example, the Box–Behnken design is used in thermoresponsive gels to achieve optimal particle stability and controlled release, making these formulations suitable for intranasal and pulmonary applications [25,68]. Such optimization tools enable the development of PLGA-based formulations with tailored characteristics, improving therapeutic outcomes in respiratory and CNS-targeted drug delivery [80,82].

The diverse techniques employed in designing PLGA-based drug delivery systems highlight the versatility and adaptability of this platform for intranasal and pulmonary applications. Methods like emulsion solvent evaporation, nanoprecipitation, spray-drying, and freeze-drying enable precise control over critical parameters such as particle size, encapsulation efficiency, and aerodynamic properties, ensuring tailored therapeutic delivery. Advanced techniques like electrospraying, microfluidics, and supercritical fluid processing further expand the potential of PLGA formulations by allowing fine-tuning of particle morphology and enhancing targeted delivery. Despite these advancements, challenges such as scalability, batch-to-batch variability, and thermal or solvent sensitivity persist, particularly for biologics and temperature-sensitive drugs. Addressing these issues through the integration of novel cryoprotectants, process optimization, and automation will be crucial for clinical translation. Additionally, optimization tools such as Box–Behnken design are valuable for parameter refinement but would benefit from the incorporation of machine learning models to accelerate development. By overcoming these technical and operational limitations, PLGA-based drug delivery systems can achieve broader clinical application and improve outcomes in complex diseases such as CNS disorders, respiratory infections, and chronic inflammatory conditions.

Table 4 outlines the methods used to prepare PLGA formulations, emphasizing their key features and suitability for encapsulating a variety of drugs. Techniques range from emulsion solvent evaporation to advanced approaches like microfluidics. The diversity of preparation techniques allows for the customization of PLGA products based on therapeutic requirements. Emulsion-based methods are widely used for encapsulating proteins and hydrophilic drugs, while spray drying and Flow Focusing^®^ ensure precise aerodynamic properties for pulmonary applications. Advanced approaches like nanoprecipitation and electrospraying support CNS and cancer therapies with stable nanoparticle formulations. Incorporating porogens enhances porosity for sustained release, while freeze drying improves stability. These methods showcase the adaptability of PLGA formulations across multiple delivery routes and therapeutic needs.

## 6. Assessing PLGA-Based Carriers in Pulmonary and Intranasal Delivery

### 6.1. Physicochemical Characterization and Morphology Analysis

The physicochemical properties of PLGA carriers, including particle size, zeta potential, surface morphology, porosity, and thermal behavior, are critical determinants of their behavior in drug delivery systems. Techniques like Dynamic Light Scattering (DLS) and Static Light Scattering (SLS) are employed to measure particle size distribution and stability, which are essential for uniformity in drug release and bioavailability [2,38,61]. For structural and surface analysis, Scanning Electron Microscopy (SEM), Transmission Electron Microscopy (TEM), and Atomic Force Microscopy (AFM) are used to visualize surface features and porosity, while Small-Angle X-ray Scattering (SAXS) can provide insights into internal nanostructures [43,76,77,90]. Fourier-Transform Infrared Spectroscopy (FTIR), Differential Scanning Calorimetry (DSC), and X-ray Diffraction (XRD) are further applied to study the crystallinity, thermal properties, and interactions between PLGA and encapsulated drugs [12,38,43,92,94]. Zeta potential measurements are critical for evaluating colloidal stability and mucoadhesive potential [13,43,87,94].

### 6.2. Biodegradability, Biocompatibility, and Cytotoxicity Studies

The safety and compatibility of PLGA formulations are vital for intranasal and pulmonary applications. Biodegradability is assessed through non-enzymatic hydrolytic degradation studies, which evaluate polymer breakdown under physiological conditions [1,10,13]. Cytotoxicity testing is conducted on various cell lines, such as Calu-3 and RPMI 2650, using MTT assays to confirm their biocompatibility [4,20,62]. Additionally, histopathological analysis provides insights into tissue response, toxicity, and inflammation after administration [51,99]. For pulmonary formulations, systemic toxicity and acute hemolysis studies are crucial for understanding the safety profile [29,37,56]. These tests ensure that PLGA carriers degrade into biocompatible byproducts without eliciting adverse effects.

### 6.3. Drug Loading, Release Kinetics, and Sustained Delivery

PLGA-based systems are optimized for efficient drug encapsulation and controlled drug release. Encapsulation efficiency is measured using techniques like High-Performance Liquid Chromatography (HPLC) or UV-Vis Spectroscopy, ensuring maximum drug loading in the carrier [6,39,97]. In vitro drug release profiling is conducted in simulated physiological fluids, such as PBS, simulated lung fluid (SLF), or nasal electrolyte solution (SNES), to evaluate sustained and biphasic release patterns [6,92,94]. Mathematical models such as the Weibull or Higuchi models are used to study release kinetics [9,16,28,30,49,65]. Stability testing under different environmental conditions is also performed to assess drug leakage and long-term integrity [12,13,30].

### 6.4. Aerodynamic Properties and Pulmonary Delivery

For pulmonary drug delivery, the aerodynamic behavior of PLGA formulations is critical. Parameters like Mass Median Aerodynamic Diameter (MMAD) and Fine Particle Fraction (FPF) are measured using Cascade Impaction Testing or the Anderson Cascade Impactor (ACI) to ensure effective lung deposition [34,43,45,46,52,54,77]. Pulmonary deposition and retention are studied through in vivo and ex vivo models, confirming the formulations’ targeting efficiency in the respiratory tract [58,92,101]. Additionally, nebulization studies evaluate the stability and compatibility of PLGA formulations with aerosolization devices [45,46,54].

### 6.5. Mucoadhesion and Nasal Permeation Studies

Mucoadhesion and permeability are key for intranasal delivery systems. Mucoadhesive properties are evaluated using polymers like chitosan through mucin particle assays or adhesion studies with nasal mucosa [42,69,94]. Ex vivo permeation studies, often conducted on sheep or goat nasal mucosa, assess drug absorption efficiency and permeability across biological barriers [25,68,87]. Gamma scintigraphy is used to study nasal clearance and residence time, ensuring prolonged retention at the site of action [69,87]. These studies are crucial for enhancing bioavailability and nasal-to-brain transport efficiency.

### 6.6. Cellular Uptake, Targeting, and Biodistribution

Understanding the cellular uptake and biodistribution of PLGA nanoparticles is essential for targeted delivery. Fluorescence microscopy and flow cytometry are employed to study nanoparticle internalization in epithelial and immune cells [3,44,75]. Biodistribution studies using radiolabeled or fluorescently tagged particles confirm CNS or pulmonary targeting [61,83,84]. Techniques like integrin receptor binding assays and endosomal escape studies are also conducted to enhance specificity for tumor or immune cells [57,62,79].

### 6.7. Immunogenicity and Immune Response Studies

For vaccine and immune-targeted therapies, systemic and mucosal immune responses are evaluated. ELISA is used to measure IgG and sIgA titers in serum and mucosal fluids, assessing the effectiveness of intranasal immunizations [70,72,76,95]. Cytokine profiling, such as IFN-gamma and IL-2 analysis, helps determine Th1/Th2 immune response balance [88,100,101]. Long-term antibody monitoring post-vaccination provides insights into the durability of the immune response [56,74,87].

### 6.8. Therapeutic Efficacy in Disease Models

PLGA formulations are tested in specific disease models to evaluate their therapeutic efficacy. For pulmonary fibrosis, antifibrotic activity is assessed by measuring TGF-beta levels, collagen deposition, and ROS scavenging in bleomycin or paraquat-induced models [49,50,51,99]. In neurodegenerative disorders, behavioral and histological assessments in Alzheimer’s and Parkinson’s models are used to evaluate neuroprotection [62,67,96]. Antitumor efficacy is studied using glioblastoma and melanoma models through apoptosis assays and tumor growth inhibition [44,61,63].

### 6.9. Pharmacokinetics and Toxicity Studies

Pharmacokinetic profiling involves assessing drug absorption, distribution, metabolism, and clearance. These studies use liquid chromatography-tandem mass spectrometry (LC-MS/MS) to measure drug levels in plasma and tissues [40,41,65]. Toxicity assessments include acute and chronic toxicity evaluations, lung histology, and systemic inflammatory marker analysis to ensure safety during administration [51,52,99].

### 6.10. Imaging and Visualization Techniques

Advanced imaging methods provide insights into nanoparticle behavior and distribution. Confocal microscopy, fluorescence molecular tomography, and quantum dot imaging allow for real-time tracking of nanoparticles in vivo [75,83,96]. Electron microscopy techniques, including TEM and SEM, visualize ultrastructural features, while CT and MRI scans assess drug localization and therapeutic effects [10,26,49].

### 6.11. Advanced Fabrication Techniques and Optimization

The preparation of PLGA nanoparticles involves methods like emulsion-solvent evaporation, nanoprecipitation, and spray drying. Optimization techniques such as Box–Behnken Design and factorial design are used to refine formulation parameters [38,59,82,91]. Surface modifications with amphiphilic polymers or ligands enhance targeting and stability [46,49,53,91].

The evaluation of PLGA-based carriers for pulmonary and intranasal drug delivery involves a comprehensive range of characterization methods, safety assessments, and therapeutic efficacy studies. While techniques such as DLS, SEM, and FTIR provide detailed insights into particle properties and drug-polymer interactions, their scalability remains a challenge for industrial applications. Similarly, drug release kinetics and aerodynamic studies are critical for optimizing delivery profiles, but the in vitro models used often fail to fully replicate in vivo conditions. Innovative tools like organ-on-a-chip systems and physiologically relevant release models could bridge this gap. In addition, the biodegradability and biocompatibility of PLGA are well-established; however, variability in degradation rates and potential immune responses underscore the need for tunable and patient-specific formulations. Advanced imaging techniques and predictive pharmacokinetic models offer great promise for improving targeting, biodistribution, and clinical translation.

Table 5 summarizes the evaluation methods for PLGA products, covering in vitro and in vivo testing, pharmacokinetics, immunogenicity, and safety analyses. It ensures the formulations meet therapeutic and regulatory requirements. Comprehensive testing confirms PLGA’s efficacy and safety. In vitro studies evaluate sustained release, mucoadhesion, and drug loading, while in vivo pharmacokinetics ensure therapeutic efficacy and bioavailability. Techniques like SEM and XRD analyze particle morphology and stability. Immunological testing confirms vaccine potency, while advanced imaging tracks biodistribution. Cytotoxicity assays and histopathological evaluations validate biocompatibility. The focus on specific disease models and delivery routes highlights PLGA’s precision in addressing diverse therapeutic challenges, from infections to gene therapy.

## 7. Proven Benefits of Using PLGA in Intranasal and Pulmonary Drug Delivery

### 7.1. Sustained and Controlled Drug Release for Chronic Diseases

PLGA-based drug delivery systems provide sustained and controlled release, a feature essential for managing chronic respiratory diseases such as cystic fibrosis, lung cancer, tuberculosis, and asthma. By maintaining continuous drug levels over extended periods, these formulations significantly reduce the frequency of dosing, thereby improving patient adherence. For example, lung cancer therapies have demonstrated efficacy lasting up to 21 days, while formulations for cystic fibrosis offer therapeutic effects for as long as a month, reducing the burden of regular administration [1,8,14,23,28,50,51,53,92]. This capability to prolong drug action without compromising efficacy establishes PLGA as a critical tool in chronic respiratory disease management.

### 7.2. Advancing Pulmonary Fibrosis Treatments

In the context of pulmonary fibrosis, PLGA nanoparticles have shown significant promise. Simvastatin-loaded PLGA nanoparticles administered via inhalation significantly attenuated paraquat-induced pulmonary fibrosis in rats. At an optimized dose of 10 mg/kg, these nanoparticles reduced inflammation, fibrosis, and serum inflammatory markers, with efficacy far surpassing that of free simvastatin [51]. Similarly, nintedanib-loaded PLGA microparticles in a bleomycin-induced murine model of idiopathic pulmonary fibrosis demonstrated improved bioavailability and sustained release over seven days, reducing inflammation and fibrosis and offering a viable option for decreasing dosing frequency in chronic lung diseases [57]. These examples highlight the clinical relevance of PLGA-based formulations in overcoming pharmacokinetic limitations and improving therapeutic outcomes.

### 7.3. Enhanced Lung Deposition and Retention

Tailored designs of PLGA particles enhance deposition and retention in the lungs, enabling efficient targeting of diseased areas while minimizing systemic exposure. Innovations in particle design, such as porous or large microparticles, allow deeper lung deposition, effectively reaching the bronchi and alveoli. Modifications like chitosan-coating further enhance mucoadhesive properties, increasing particle residence time and ensuring that drugs like calcitonin remain localized at therapeutic sites within the lungs [5,21,34,35,49,57,81,82]. This precision targeting boosts treatment efficacy and reduces off-target effects, making PLGA highly beneficial for localized respiratory therapies.

### 7.4. Biocompatibility and Long-Term Safety

PLGA’s biocompatibility and its degradation into non-toxic byproducts make it safe for prolonged use in pulmonary and nasal applications. Formulations have demonstrated stable drug release with minimal cytotoxicity, even for potent compounds such as doxorubicin and lysozyme, which show reduced toxicity and inflammation in lung models [2,7,17,29,34,37,38]. The gradual breakdown of PLGA into lactic and glycolic acids, easily metabolized by the body, further supports its safety for long-term use, especially in therapies requiring sustained local release of potentially cytotoxic agents [1].

### 7.5. Expanding Systemic Applications Through Pulmonary Delivery

In addition to respiratory applications, PLGA formulations are paving the way for treating systemic diseases via pulmonary delivery. For example, large-porous particles loaded with deslorelin achieve systemic drug levels sustained over a week, suggesting that pulmonary delivery could provide a non-invasive alternative for managing conditions like hypertension and hormone deficiencies [19,86]. This innovative approach broadens the scope of PLGA applications and offers a practical alternative to traditional systemic administration.

### 7.6. Non-Invasive CNS Delivery via Intranasal Route

The ability of PLGA nanoparticles to bypass the blood–brain barrier through intranasal administration marks a significant advancement in treating central nervous system (CNS) disorders. Non-invasive intranasal delivery has shown promise for Alzheimer’s and Parkinson’s diseases, with modifications like lactoferrin, RVG29, and mannose-enhancing drug accumulation in the brain [3,11,12,25,66,83,102]. For instance, tumor necrosis factor (TNF)-related apoptosis-inducing ligand (TRAIL)-neutralizing antibodies adsorbed onto PLGA nanoparticles, when administered intranasally, significantly enhanced brain targeting and reduced inflammation in an Alzheimer’s mouse model, thereby improving cognitive outcomes [67]. Similarly, galantamine-loaded PLGA nanoparticles successfully targeted the hippocampus when delivered intranasally, mitigating Alzheimer’s-related damage and representing a promising alternative to systemic administration [93]. Such targeted delivery minimizes systemic exposure and side effects, demonstrating the potential of PLGA for addressing complex CNS conditions.

### 7.7. Reduced Side Effects and Enhanced Therapeutic Efficacy

PLGA formulations designed for CNS and pulmonary applications enhance therapeutic efficacy while reducing side effects. CNS-targeted drugs like ropinirole, oxcarbazepine, and haloperidol, when delivered via PLGA, exhibit improved brain uptake, enabling lower doses and reducing adverse effects [13,96,103]. Similarly, in pulmonary applications, PLGA-loaded anti-fibrotic agents such as thymoquinone and simvastatin have shown promise in reducing lung fibrosis and inflammation in chronic disease models [50,51]. These sustained-release formulations are particularly valuable for patients requiring long-term management of neurological and respiratory conditions.

### 7.8. Targeted Oncology Therapies

In oncology, PLGA-based systems have shown efficacy in glioblastoma and lung cancer. Temozolomide hexadecyl ester-loaded PLGA nanoparticles, administered intranasally, overcame drug resistance, significantly prolonged survival, and reduced tumor progression in a mouse model. This formulation demonstrated the ability to bypass systemic toxicity and enhance therapeutic outcomes in glioblastoma [63]. Similarly, doxorubicin-loaded PLGA microparticles, optimized for pulmonary delivery, reduced tumor lesions and extended survival in a melanoma lung metastasis model, providing a translational pathway for PLGA systems in lung cancer therapy [47].

### 7.9. Enhanced Stability and Bioavailability

By incorporating stabilizers like PVA and PEG, PLGA-based systems enhance drug stability and bioavailability, ensuring consistent therapeutic effects for sensitive drugs such as peptides and small molecules. For instance, docetaxel-loaded PLGA particles with cryoprotective properties have maintained the stability of this potent cancer drug throughout storage and administration [25,32]. Such stability improvements ensure that therapeutic agents remain effective in challenging environments, further underscoring PLGA’s reliability as a delivery platform.

### 7.10. Expanding Delivery Applications with Surface Modifications

Surface modifications such as chitosan, mannose, and polysorbate 80 improve mucoadhesion, targeting, and permeation across mucosal barriers, making PLGA a versatile platform for pulmonary and CNS applications. Polysorbate 80-coated PLGA nanoparticles, for instance, increase brain uptake of gabapentin, while chitosan-modified PLGA enhances ropinirole delivery across nasal mucosa, effectively targeting the CNS [13,64,66]. These multifunctional coatings expand PLGA’s applications, enabling effective delivery across various biological barriers.

### 7.11. Combination Therapies and Multifactorial Disease Management

The ability of PLGA to encapsulate multiple drugs has facilitated the development of combination therapies, particularly for diseases requiring multi-drug regimens. Mannose-coated PLGA nanoparticles co-loaded with donepezil and memantine have shown improved efficacy in Alzheimer’s treatment, while dual-drug formulations combining curcumin and cisplatin demonstrate enhanced outcomes in pediatric glioma [10,26,61,66]. This flexibility in formulation enables PLGA-based systems to address the complex therapeutic needs of multifactorial diseases effectively.

### 7.12. Advances in Cancer and Vaccine Applications

PLGA enhances cancer therapies by enabling controlled, targeted release of anticancer agents, improving therapeutic outcomes. Co-loaded formulations of doxorubicin and paclitaxel for lung cancer, and curcumin and cisplatin for brainstem glioma, have demonstrated synergistic anti-tumor effects and prolonged survival in preclinical models [10,26,27,43,47,61]. The ability to precisely target tumor sites while minimizing systemic exposure underscores PLGA’s value in treating aggressive cancers. In vaccine delivery, PLGA particles have proven highly effective as carriers and adjuvants, stimulating robust mucosal and systemic immune responses. Formulations containing malaria and Hepatitis B antigens elicit strong Th1 and Th2 responses with elevated IgA and IgG levels, positioning PLGA as a leading platform for mucosal vaccine development [69,87,88]. This capability to generate durable immune responses highlights PLGA’s potential for tackling a range of pathogens.

### 7.13. Gene Therapies and RNA-Based Therapeutics

Advances in PLGA formulations, especially when combined with PEI and lipid-core modifications, have enabled effective delivery of DNA and siRNA for pulmonary gene therapies. These nanoparticles achieve high gene expression and T-cell activation, showing promise in treating genetic lung diseases and enhancing pulmonary immunization strategies [75,78]. PLGA’s contributions to non-viral gene delivery represent a significant step forward for RNA-based therapeutics.

### 7.14. Prolonged Efficacy in Chronic Conditions

The sustained-release properties of PLGA formulations provide long-lasting therapeutic effects, reducing dosing frequency and maximizing local efficacy for chronic conditions like lung cancer and fibrosis. For instance, doxorubicin and artesunate-loaded PLGA particles deliver prolonged anti-tumor activity, supporting the management of diseases requiring sustained intervention [43,47]. PLGA’s ability to maintain extended therapeutic effects underscores its utility in chronic disease treatment.

PLGA-based systems have demonstrated remarkable versatility in intranasal and pulmonary drug delivery, offering benefits such as sustained drug release, enhanced targeting, and reduced systemic toxicity. These formulations address critical challenges in treating chronic diseases, CNS disorders, and cancers, while also showing promise in vaccine delivery. However, issues such as variability in polymer degradation, patient-specific physiological differences, and challenges in scaling up production remain barriers to clinical translation. Future efforts should focus on developing predictive models, refining particle designs, and integrating patient-specific delivery strategies to maximize therapeutic outcomes.

Table 6 identifies the benefits of PLGA formulations, including sustained release, high encapsulation efficiency, biocompatibility, and the ability to target specific diseases with minimal side effects. PLGA offers sustained drug release, enhancing patient compliance and reducing dosing frequency. Its high encapsulation efficiency ensures the effective delivery of hydrophilic and hydrophobic drugs. Customizable formulations allow disease-specific therapies, from cancer to neurodegenerative disorders. Advanced targeting mechanisms reduce side effects and enhance therapeutic efficacy. Biocompatibility ensures safety for long-term applications. The versatility of PLGA formulations supports co-delivery systems and synergistic effects in multi-drug therapies, making it a cornerstone in modern drug delivery systems.

## 8. Concluding Remarks

Poly(D,L-lactic-co-glycolic acid) (PLGA) has firmly established itself as a transformative material in drug delivery, offering unique advantages such as sustained release, biocompatibility, and biodegradability. Its versatility has revolutionized therapeutic approaches for challenging conditions, including chronic respiratory diseases, CNS disorders, and complex cancers. Through precise drug targeting, enhanced bioavailability, and reduced systemic toxicity, PLGA-based systems have opened new avenues in localized and systemic therapies.

However, while the field has made remarkable progress, there remain critical challenges that need to be addressed to realize the full clinical potential of PLGA systems. Achieving reliable and tunable drug release profiles, refining particle designs for enhanced targeting and bioavailability, and ensuring scalability in manufacturing processes are pressing priorities. Additionally, long-term safety evaluations and immunogenicity assessments must be expanded, particularly for chronic and repeated-use therapies, to mitigate potential risks.

Looking ahead, researchers in the field have an opportunity to build upon existing innovations by embracing emerging technologies and collaborative approaches. Advanced techniques, such as pH-sensitive or enzyme-responsive formulations, microfluidics, and 3D printing, can significantly improve particle customization and therapeutic precision. The integration of computational tools, including machine learning and predictive modeling, can further accelerate formulation optimization and reduce the time and cost of clinical translation.

Ultimately, the future of PLGA-based drug delivery lies in interdisciplinary collaboration, where materials science, bioengineering, and computational approaches converge to overcome existing limitations. By driving innovation and refining formulation strategies, researchers can unlock the immense potential of PLGA, advancing the field toward more effective, safer, and accessible therapies for a wide range of diseases. This path forward not only ensures the continued evolution of PLGA systems but also positions them as a cornerstone of next-generation drug delivery platforms.

## Figures and Tables

**Table 1:** Therapeutic agents delivered via PLGA systems: indications and benefits.

Drug(s)	Therapeutic Purpose	References
Levofloxacin, Tobramycin, Ciprofloxacin, Azithromycin	Treatment of bacterial infections, including pulmonary infections, biofilm penetration, cystic fibrosis, and chronic lung diseases.	[9,15,23,24,28,29,31,45]
Insulin	Prolonged pulmonary delivery for diabetes management with enhanced hypoglycemic effects and optimized formulations.	[35,36,46]
Celecoxib, Docetaxel, Paclitaxel	Lung cancer treatment with enhanced tumor targeting, sustained delivery, synergistic effects, and reduced side effects.	[8,10,26,27,47]
Alpha-mangostin, Rosmarinic acid, Simvastatin, Thymoquinone, Nifedipine	Treatment of pulmonary fibrosis and inflammation, reducing oxidative stress, and improving lung function.	[17,48,49,50,51,52]
Ethionamide, Rifampicin, Rifapentine, Linezolid	Pulmonary tuberculosis therapy with targeted drug release, prolonged lung retention, and macrophage-targeted delivery.	[30,53,54,55,56]
Recombinant human interleukin-2 (rhIL-2)	Pulmonary delivery of cytokines for immune modulation, retained bioactivity, and therapeutic protein release.	[33,39]
Nintedanib, Pirfenidone	Anti-fibrotic agents for treating idiopathic pulmonary fibrosis with prolonged lung retention and reduced fibrosis progression.	[33,49,57]
Sildenafil citrate, Tadalafil, Prostaglandin E1	Pulmonary arterial hypertension management with improved bioavailability, reduced toxicity, and extended drug release.	[16,18,19,58,59,60]
Doxorubicin, Artesunate, Oridonin, Curcumin, Temozolomide	Pulmonary and CNS cancer therapies with enhanced tumor targeting, reduced tumor hypoxia, and prolonged survival rates.	[10,43,44,61,62,63]
Vitamin D3, Amiodarone, Gabapentin, Topiramate	Neurological and pulmonary therapies with improved systemic delivery, brain targeting, and reduced toxicity.	[7,25,41,64,65]
Rotigotine, Donepezil, Memantine, Huperzine A, Gabapentin, Ropinirole Hydrochloride (RH)	CNS disorders such as Parkinson’s and Alzheimer’s disease with improved brain targeting, therapeutic efficacy, and bioavailability.	[12,13,40,62,66,67]
Favipiravir	Treatment of viral infections such as COVID-19 via enhanced nasal delivery systems and sustained release.	[32,68]
Deslorelin, Alpha-1 antitrypsin, Calcitonin, Somatropin	Sustained release for systemic and pulmonary hormone replacement, protein therapies, or calcium regulation.	[6,19,21,38]
Vaccines (e.g., SPf66 malaria, BPI3V, CNA19, HBsAg)	Nasal and pulmonary vaccine delivery with robust mucosal and systemic immune responses and optimized antigen stability.	[22,69,70,71,72,73]
DNA/RNA Therapeutics (e.g., FMDV DNA, siRNA)	Gene therapy for respiratory diseases, with high transfection efficiency, targeted delivery, and enhanced immune responses.	[74,75,76,77,78,79]

**Table 2:** Key materials supporting PLGA formulations.

Polymers and Excipients	Key Patterns and Applications	References
PLGA, PEG-PLGA, Chitosan, PVA, Mannitol, Sorbitol, Lactose	Biodegradable and biocompatible systems for sustained drug release, enhanced bioavailability, and reduced cytotoxicity.	[1,2,5,16,28,36,39]
PLGA, Chitosan, DPPC, Leucine, Cyclodextrins	Enhanced mucoadhesion and nasal or pulmonary delivery with controlled particle size and drug release properties.	[11,20,34,68,81,87]
PLGA, Chitosan, TMC, Glycol Chitosan, Lactoferrin	Improved systemic and CNS delivery, enhanced brain targeting for neurodegenerative and CNS disorders.	[3,12,13,25,40,62]
PLGA, PEG, PVA, Poloxamer, Poloxamine	Tunable aerodynamic and physical properties for pulmonary drug delivery.	[4,6,16,60,75,82]
PLGA, PEI, Poloxamer, Tween 20	Efficient gene and siRNA delivery systems with high transfection efficiency and targeted gene therapy potential.	[74,75,78,79]
PLGA, QS-21, CpG-ODN, Chitosan	Effective vaccine delivery with robust systemic and mucosal immune responses.	[56,73,85,87,88]
PLGA, Amphiphilic Block Copolymers, Borneol	Multifunctional drug carriers for localized therapy and enhanced therapeutic efficacy.	[10,26,27,77]
PLGA, PVA, Chitosan, Kolliphor	Anti-inflammatory and antimicrobial applications in pulmonary therapies.	[7,9,15,45]
PLGA, DPPC, Sorbitol, Leucine, Cyclodextrins	Pulmonary fibrosis treatment with enhanced therapeutic effects and reduced inflammation.	[48,49,50,52]
PLGA, Chitosan, PVA, Lactose	Mucoadhesive formulations for effective nasal vaccine delivery.	[22,72,76,89]
PLGA, PEI, PVA, Lactose	DNA and RNA delivery platforms with potential applications in pulmonary and nasal gene therapy.	[75,77,78]

**Table 3:** Core properties of PLGA systems for optimized drug delivery.

Physicochemical Properties	Description	References
Sustained Drug Release	Controlled and prolonged release ranging from hours to weeks for localized or systemic delivery.	[1,2,10,16,19,25,36,39,59,92]
High Encapsulation Efficiency	Achieved >70% efficiency for diverse drugs, maintaining stability and preventing degradation.	[10,17,25,31,36,37,39,53,59,85,92]
Particle Size Control	Tunable sizes from nanoscale (<200 nm) to microscale (1–20 µm) for specific delivery needs.	[4,22,25,34,35,54,57,65,81]
Surface Charge Modifications	Cationic and anionic modifications to enhance mucoadhesion, uptake, or lung retention.	[3,4,11,20,74,76]
Aerodynamic Properties for Pulmonary Delivery	Optimized aerodynamic diameters (1–5 µm) and low density for deep lung deposition.	[5,14,16,25,37,58,82]
Porosity and Surface Morphology	Porous/dimpled particles enhance drug release, retention, and avoid macrophage uptake.	[5,8,19,27,34,58,69]
Biodegradability and Biocompatibility	Safe degradation into lactic and glycolic acid; no cytotoxic effects; suitable for long-term use.	[1,2,5,6,7,22]
Colloidal Stability	Formulations resist aggregation, ensuring reliable delivery and extended shelf life.	[4,29,30,31,49,70]
Targeting and Mucoadhesive Properties	Improved mucoadhesion and tissue targeting through polymers like chitosan and ligands.	[3,12,20,21,40,66,87]
Hydrophilic and Hydrophobic Drug Compatibility	Versatile drug encapsulation enabling both hydrophilic and hydrophobic drug formulations.	[9,19,28,43,54,65]
Enhanced Intracellular Uptake and Endosomal Escape	Nanoparticles designed for intracellular delivery with superior escape mechanisms.	[12,62,67,75,83,98]
Fine Particle Fraction (FPF)	High FPF for inhalation formulations, enabling effective deep lung drug delivery.	[17,35,45,54,68,81]
Drug Loading Capacity	High loading efficiency (up to 30%) for achieving therapeutic drug concentrations.	[19,34,39,52,89]
Dual Drug Delivery Systems	Co-delivery of drugs for synergistic therapeutic effects, especially for cancer and infections.	[15,26,27,71]
Controlled Degradation Profiles	PLGA formulations tailored for specific degradation rates based on polymer composition.	[1,10,19,57]
Immune Response Modulation	Surface-modified PLGA particles to enhance antigen presentation and sIgA production.	[22,69,72,73]

**Table 4:** Techniques used in PLGA-based drug delivery system development.

Preparation/Processing Technique	Key Features and Applications	References
Emulsion Solvent Evaporation	Widely used for hydrophilic and hydrophobic drugs, providing high encapsulation efficiency and size control.	[2,19,21,24,31,36,37,39,45,46,47,53,63,88]
Double Emulsion Solvent Evaporation (w/o/w)	Effective for encapsulating proteins, peptides, and hydrophilic drugs, retaining bioactivity and minimizing burst release.	[2,19,34,36,39,40,58,60,87,89]
Spray Drying	Produces low-density, fine particles for pulmonary applications with excellent aerodynamic properties.	[5,15,23,35,54,86,91,94]
Nanoprecipitation	Ideal for stable, small nanoparticles with high drug loading for nasal, pulmonary, and CNS delivery.	[7,10,11,12,13,31,32,41,62]
Freeze Drying (Lyophilization)	Stabilizes formulations, prevents aggregation, and improves storage for long-term use.	[23,24,25,30,96]
Supercritical Fluid Technology	Environmentally friendly method for preparing porous microparticles with controlled porosity and size.	[8]
Flow Focusing^®^ Technology	Produces highly uniform particles, particularly for chronic disease treatments via pulmonary delivery.	[9]
Hot-Melt Extrusion	Combined with porogens for controlled-release microparticles for pulmonary hypertension therapy.	[18]
Membrane Emulsification	Ensures uniform particle sizes with high encapsulation efficiency, particularly for hydrophobic drugs.	[49]
One-step Emulsification	Simplified process for producing porous microparticles with enhanced lung retention properties.	[5,99]
Top-down Particle Engineering	Produces discoidal particles tailored for improved lung deposition and controlled aerodynamic performance.	[57]
Surface Modification (e.g., Ligand/Chitosan Coating)	Enhances targeting, mucoadhesion, and immune response; applied in nasal, pulmonary, and vaccine delivery.	[3,20,21,22,29,40,66,78,100]
Cryoprotectant-Assisted Formulations	Stabilizes nanoparticles during freeze drying while maintaining bioactivity and redispersibility.	[32,96]
Electrospraying	Produces porous microparticles for cancer and fibrosis therapy with high drug loading and fine particle fractions.	[10]
Box–Behnken Design and Optimization	Optimizes particle size, drug release, and encapsulation efficiency for therapeutic tailoring.	[12,31,40,68,82]
Microfluidics	Advanced technique for precise shell-core nanoparticles and siRNA delivery.	[75]
Combination Technologies (e.g., Spray Drying + Nanoprecipitation)	Hybrid approaches for co-delivery systems and synergistic drug combinations.	[15,26,27,91]
Porogens for Porous Structures (e.g., Ammonium Bicarbonate)	Enhances porosity, lung retention, and controlled drug release for pulmonary applications.	[18,19,34]
Design of Experiments (DoEs)	Applied for optimizing formulations to achieve desired drug release profiles and physicochemical properties.	[31,49,57,60,68,82]

**Table 5:** Methods for assessing the performance of PLGA formulation.

Testing/Evaluation Method	Description	References
In Vitro Drug Release Studies	Evaluates release kinetics, focusing on sustained, controlled, or burst release profiles, often paired with modeling approaches.	[24,26,28,30,31,60,71,92]
Particle Size and Morphology Analysis	Uses SEM, TEM, and laser diffraction to assess uniformity, aerodynamic performance, and surface characteristics.	[5,22,35,43,54,57,97]
Encapsulation Efficiency and Drug Loading	Measures drug entrapment efficiency and loading capacity to ensure optimized therapeutic payloads.	[17,19,39,78,80]
In Vivo Pharmacokinetics and Bioavailability	Analyzes plasma drug concentration, lung retention, and bioavailability for systemic and localized therapies.	[19,28,33,51,59,65]
Mucoadhesion and Nasal Permeation Tests	Evaluates formulations’ adhesion to mucosal surfaces and their ability to permeate nasal epithelium for enhanced delivery.	[20,21,22,25,68,71]
Aerodynamic Property Testing	FPF, MMAD, and respirable fractions are measured to ensure efficient pulmonary deposition and retention.	[5,8,10,18,24,34,52,54,86]
Cytotoxicity and Biocompatibility Assays	Includes MTT assays, ROS production tests, and histopathological studies to confirm safe use in cells and tissues.	[4,7,17,62,64,75]
Cellular Uptake and Endosomal Escape	Analyzes nanoparticle internalization by cells and their escape from endosomal pathways to enhance therapeutic effects.	[11,12,67,75,83,98]
Immunological Testing for Vaccine Formulations	Measures IgA/IgG titers, cytokine responses, and mucosal/systemic immunity for evaluating vaccine efficacy.	[69,72,73,74,87,88]
Inflammatory and Oxidative Stress Marker Analysis	Reduces markers related to fibrosis, cancer, and inflammation, particularly for pulmonary and systemic diseases.	[33,49,50,51,52]
Histopathology and Tissue Analysis	Examines tissue-level toxicity and therapeutic impact using staining, imaging, and immunohistochemistry.	[7,41,43,57,65,75]
Pharmacodynamics and Therapeutic Efficacy Studies	Focuses on therapeutic outcomes such as blood glucose control, cancer inhibition, and fibrosis reduction.	[26,44,46,51,57,61]
Antibacterial and Antitumor Activity Testing	Tests bacterial killing efficiency, apoptosis induction, and tumor growth inhibition in vitro and in vivo.	[9,15,27,44,61]
Safety, Stability, and Shelf-Life Testing	Evaluates formulation stability during storage, including freeze drying and aggregation prevention.	[10,23,25,37,96]
Gene Delivery and Transfection Efficiency	Includes siRNA/DNA internalization, immune response monitoring, and cellular transfection efficacy.	[74,75,76,77,78]
Metabolic Stability and Degradation Studies	Monitors polymer degradation, metabolic stability, and retention of bioactivity during release.	[24,40,49,60,89]
Advanced Imaging and Biodistribution	Uses fluorescence imaging, CT scans, and molecular tomography to track biodistribution and targeting efficiency.	[49,62,79,83,96]
Design of Experiments (DoEs) for Optimization	Utilized to optimize particle size, encapsulation efficiency, drug release profiles, and aerodynamic properties.	[25,31,57,60,68,82]

**Table 6:** Advantages of using PLGA in nasal and pulmonary applications.

Benefit	Description	References
Sustained and Controlled Drug Release	Enables prolonged therapeutic effects, reduces dosing frequency, and improves patient compliance.	[1,2,19,24,36,39,92]
High Encapsulation Efficiency and Drug Loading	Ensures effective delivery of hydrophilic and hydrophobic drugs with minimal waste.	[10,17,18,51,53,85]
Tailored Particle Size for Specific Applications	Allows precision in pulmonary, nasal, and systemic drug delivery by optimizing aerodynamic and absorption properties.	[5,25,35,40,54,57,82]
Enhanced Bioavailability and Targeting	Improves drug delivery to specific tissues or organs, including lungs, CNS, and mucosal surfaces.	[3,12,13,21,33,62]
Biocompatibility and Safety	Biodegradable and biocompatible, with degradation into lactic and glycolic acids; non-toxic even for long-term use.	[1,5,6,7,22]
Adaptability to Complex Formulations	Compatible with co-delivery of multiple drugs, enabling synergistic effects in therapies.	[15,26,27,87]
Versatility in Preparation Methods	Can be prepared using diverse techniques such as emulsification, nanoprecipitation, spray drying, and supercritical fluids.	[2,8,9,10,49]
Immune Modulation and Vaccine Efficiency	Enhances mucosal and systemic immune responses for effective vaccination via nasal and pulmonary routes.	[22,69,72,73,88]
Improved Stability and Shelf-Life	Freeze drying and cryoprotectant-assisted techniques ensure long-term stability of formulations.	[23,24,25,96]
Enhanced Intracellular Uptake	Surface-modified nanoparticles increase cellular uptake, ensuring effective drug delivery to target cells.	[11,12,67,75,83,98]
Reduction of Side Effects	Localized drug delivery minimizes systemic toxicity, particularly in cancer, hypertension, and pulmonary therapies.	[7,18,27,59]
Effective Pulmonary Delivery	Optimized aerodynamic properties and low-density formulations enhance lung deposition and retention.	[5,14,15,16,60]
Support for Gene Therapy and siRNA Delivery	High transfection efficiency and stability support the development of genetic therapies.	[74,75,77,78]
Antioxidant and Anti-Inflammatory Benefits	Encapsulation of bioactive compounds like curcumin and simvastatin reduces oxidative stress and inflammation.	[17,49,50,51,62]
Potential for Disease-Specific Customization	Customizable formulations address diseases such as tuberculosis, diabetes, cancer, fibrosis, and CNS disorders.	[28,33,36,47,57]
